# Innovative photobiomodulation-enhanced T-PRF in posterior mandibular fractures: a randomized pilot study

**DOI:** 10.1186/s12903-026-08455-8

**Published:** 2026-05-09

**Authors:** Amr Hassaan Elyamany, Mona S. Oraby, Marwa G. Noureldin

**Affiliations:** Department of Oral and Maxillofacial Surgery, Faculty of Dentistry, Alexandria University, Alexandria, Egypt

**Keywords:** Low-level laser therapy (LLLT), Titanium-prepared platelet-rich fibrin (T-PRF), Mandibular fractures, Bone regeneration, Bone density

## Abstract

**Background:**

Mandibular body fractures are common maxillofacial injuries, often resulting from road traffic accidents. Optimal management seeks anatomical reduction and stable fixation. Recent advances highlight the adjunctive use of low-level laser therapy (LLLT) and Titanium-Prepared Platelet-Rich Fibrin (T-PRF) to enhance bone healing. LLLT stimulates osteogenesis and angiogenesis, while T-PRF provides sustained growth factor release. This pilot study examines the synergistic potential of LLLT and T-PRF in improving bone regeneration and clinical outcomes in mandibular body fracture repair.

**Patients and methods:**

Open reduction and internal fixation (ORIF) of posterior mandibular fractures was performed using titanium plates and screws. Patients were divided into two groups: the control group received T-PRF placement at the fracture site, while the study group received T-PRF followed by postoperative LLLT sessions. Using Computerized Tomography, bone mineral density was evaluated at three months postoperatively. Clinical parameters, including pain, wound healing, maximum mouth opening, and edema, were monitored postoperatively to evaluate recovery dynamics.

**Results:**

Twelve patients (14 fracture lines) were treated with ORIF and divided equally into two groups. All patients achieved satisfactory anatomical reduction and stable occlusion. The study group demonstrated significantly higher bone density at 12 weeks and experienced significantly greater pain reduction during the first and second weeks postoperatively (p < 0.05) compared to controls. Both groups showed similar results in mouth opening, wound healing, and edema resolution, with no significant intergroup differences in these parameters.

**Conclusion:**

The adjunctive application of LLLT with T-PRF significantly enhanced osseous regeneration in the study group, yielding higher bone density at 12 weeks and greater pain reduction compared to T-PRF alone. However, both groups demonstrated equivalent improvements in clinical outcomes - including edema resolution, maximum mouth opening, and wound healing - with no significant intergroup differences.

## Introduction

Mandibular fractures constitute a significant proportion of facial trauma, accounting for approximately 25% of all maxillofacial fractures [1, 2]. These injuries predominantly affect young adult males, with the highest incidence observed in individuals aged 21 to 30 years. Road traffic accidents are the primary cause, responsible for approximately 62.8% of cases, followed by sports-related injuries, assaults, falls, and complications arising from dental extractions [3]. Studies report that fractures of the mandibular body comprise about 29% of all mandibular fractures, with incidence rates ranging from 11% to 36%. This is followed in frequency by fractures of the condyle and the angle [1].

The primary goals in the management of mandibular fractures are to restore the patient’s pre-injury function and aesthetics by achieving accurate anatomical reduction and stable fixation of the fracture fragments. Central to this process is the reestablishment of normal occlusion, which ensures proper alignment of the dental arches and supports facial symmetry. Effective immobilization of the fracture segments promotes direct bone healing and minimizes the risk of complications, thereby facilitating a return to normal masticatory function and jaw strength. Ultimately, the treatment aims to achieve both functional and aesthetic rehabilitation with the least possible disability and shortest recovery period 4, 5 [6].

Surgical management of mandibular fractures often requires internal fixation to stabilize bone segments, promote osseous union, and restore both functional integrity and facial aesthetics. A range of fixation devices is utilized for mandibular stabilization, including standard fixation plates, locking plates, and reconstruction plates. Specific applications involve Champy’s miniplates [7], lag screws, three-dimensional (3D) plates, twin fork miniplates, sliding plates, and V-shaped plates. The materials used in the fabrication of these devices include polyglycolic acid and polylactic acid polymers, carbon-reinforced polymers, and titanium alloys [8].

The medical community has developed a robust understanding of modulating cellular function through chemical entities or macromolecular agents, such as growth factors, genetic material, or their components. However, the ability to modify biological target activity via physical modalities remains a more contemporary and less explored research domain. Initially, studies prioritized energy absorption capacity in biological systems, focusing on dosage as the primary determinant of therapeutic effects. Current evidence now recognizes biophysical stimulation as a valid therapeutic strategy to activate osteogenesis and accelerate recovery [9].

Low-level laser therapy (LLLT), or photobiomodulation, effectively enhances osseous fracture repair by modulating cellular processes critical to bone healing. LLLT stimulates osteoblast proliferation and activity, increases collagen synthesis, and upregulates key osteogenic transcription factors and growth factors such as Runx2 and Bone Morphogenetic Protein-2 (BMP-2). Additionally, it exerts anti-inflammatory effects and promotes angiogenesis, creating a favorable microenvironment for bone regeneration. Preclinical and clinical studies demonstrate that LLLT significantly improves bone mineral density, accelerates callus formation, and enhances fracture consolidation rates, highlighting its potential as a valuable adjunct in managing skeletal injuries [10, 11].

Titanium-Prepared Platelet-Rich Fibrin (T-PRF) is a novel autologous grafting material characterized by elevated concentrations of platelets and leukocytes, which act as reservoirs for key growth factors such as platelet-derived growth factor (PDGF), transforming growth factor-beta (TGF-β), vascular endothelial growth factor (VEGF), and epidermal growth factor (EGF). Preparation of T-PRF using titanium tubes is hypothesized to enhance platelet activation and reduce the risk of silica particle contamination compared to traditional glass tubes, where friction may introduce suspended silica particles. This method results in a denser and more organized fibrin network, with greater structural integrity and a larger fibrin area than conventional PRF, leukocyte-platelet-rich fibrin (L-PRF). These properties are believed to contribute to T-PRF’s regenerative potential, promoting effective bone regeneration, periodontal healing, and improved wound healing [12].

The synergistic application of biomaterial scaffolds and low-level laser therapy combines their respective advantages to optimize bone regeneration. Scaffolds provide a 3D structural framework that supports osseous tissue ingrowth, while LLLT modulates critical cellular repair mechanisms. Research demonstrates that LLLT enhances osteoblast proliferation and functional activity within scaffold matrices, promotes vascular network development, and accelerates mineralized tissue deposition [13, 14]. This synergy improves scaffold integration and bone-healing outcomes, particularly in complex fractures requiring structural and biological support.

Although both LLLT and T-PRF are known to aid bone healing in mandibular fractures, their combined effect has not been thoroughly studied. This preliminary study aims to compare T-PRF alone versus T-PRF with LLLT to see if the combination offers additional benefits. The null hypothesis states that adding LLLT to T-PRF does not significantly improve bone healing or clinical outcomes compared to T-PRF alone.

## Patients and methods

### Aim of the study

To compare the effect of using T-PRF versus T-PRF with Low Level Laser stimulation on bone healing at posterior mandibular fractures.

#### Study design

This study is a prospective, randomized pilot study conducted with adherence to the CONSORT guidelines [15, 16]. The study adhered to the ethical principles of the Declaration of Helsinki for human research and received prior approval from the Research Ethics Committee of the Faculty of Dentistry, Alexandria University (IRB: 0855-02/2024) and was retrospectively registered on 2025-05−13 at the official ClinicalTrials.gov^1^ as “T-prf Versus T-prf and Low-level Laser Stimulation on Bone Healing at Posterior Mandibular Fractures” (identifier: NCT06970379).

### Study flow chart

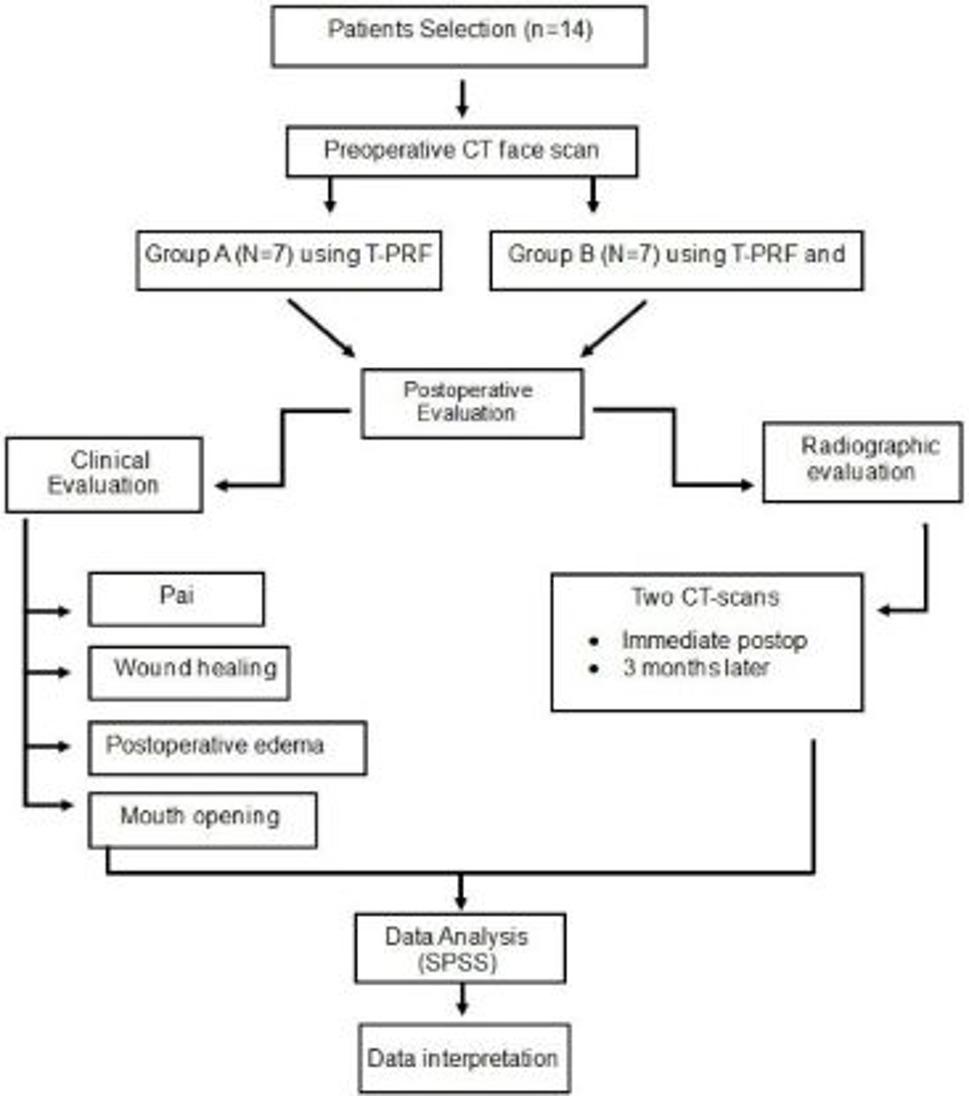



### Study setting and location

Patients with recent mandibular fractures were selected from those admitted to the Emergency Department of Alexandria University Hospital from March 2023 to January 2025. Surgical interventions were performed at the Department of Oral and Maxillofacial Surgery, Faculty of Dentistry, Alexandria University. All participants provided written informed consent following a comprehensive explanation of the surgical procedures, including potential benefits and risks.

### Sample size estimation

Sample size was estimated assuming 5% alpha error and 80% study power using Rosner’s method^2^ and G-Power software version 3.1.9.7^3^, assuming a 5% alpha error and 80% statistical power. Srinivas et al. stated that the mean increase in bone density after 3-months was estimated to be 244.97 ± 95.16 after applying PRF [17]. In parallel, Zaky et al. reported a 45.825 ± 11.06 Gy scale points (almost equivalent to 156.86 ± 57.24 HU) increase in bone density in 3 months after applying LLLT to promote bone healing [18]. Thus, it was assumed that the mean increase in bone density after applying PRF in addition to LLLT would be 401.83 with a pooled SD of 76.20. Based on a comparison of means, the required sample size per group is 6, which will be increased to 7 to make up for the loss to follow-up. The total sample size = number per group x number of groups = 7 × 2 = 14 patients.

### Criteria for patient selection

#### Inclusion criteria

- Adult subjects aged 20 to 40 years, with no gender restrictions.
- Participants presented with a recent, non-infected mandibular angle fracture requiring open reduction and internal fixation.

#### Exclusion criteria

- Pre-existing hematological disorders (such as thrombocytopenia and hemorrhagic diseases).
- Patients with diabetes mellitus.
- Old chronic or infected fractures.
- Fractures resulting from pathological conditions.

### Randomization

The participants were randomly allocated to two treatment groups via computer-generated randomization with a 1:1 allocation ratio [19].

### Grouping of the patients

- Control group (*n* = 7) received T-PRF alone.
- Study group (*n* = 7) received a combination of T-PRF and LLLT.

## Methods

### Pre-surgical assessment and preparation

#### History

Comprehensive personal information, including the patient’s name, age, gender, occupation, contact number, and address, was collected. A detailed medical history, encompassing any past or recent illnesses, was documented. Trauma-related data were obtained, including the nature, cause, date, and location of the incident.

#### General examination

A general physical examination was performed to assess associated injuries, soft tissue lacerations, level of consciousness, active bleeding, and any initial first aid administered. This was followed by a thorough intraoral and extraoral examination using both inspection and palpation techniques. Clinical findings such as swelling, facial asymmetry, bleeding, soft tissue injuries, malocclusion, and jaw misalignment during movement were recorded. Additional signs, including buccal ecchymosis and lingual hematoma, were noted when present. Segmental mobility and any evidence of anesthesia or paresthesia were also assessed.

#### Radiographic examination

**Fig. 1:**
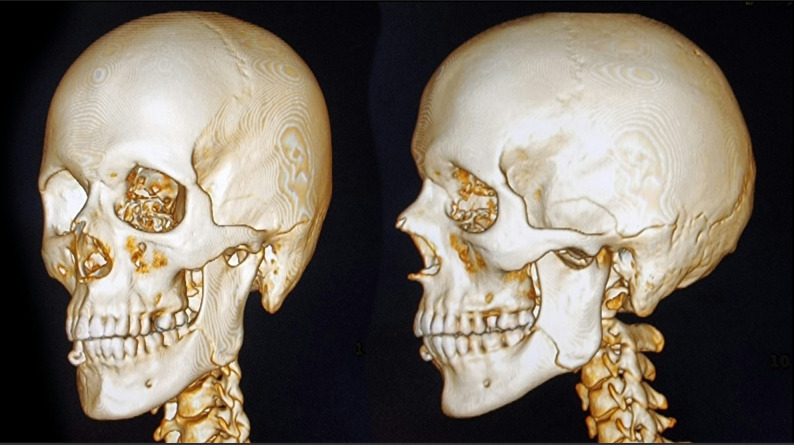
**3D window of preoperative CT view showing left mandibular angular fracture.**

Computed tomography (CT) scans were performed to evaluate the number and configuration of fracture lines, the degree of displacement, and the involvement of teeth in the fracture (Fig. 1).

#### Patient preparation

All necessary laboratory investigations were conducted to obtain anesthesiologist clearance prior to surgery. To prevent postoperative infections, patients received an intravenous dose of 1 gram of cefotaxime (Cefotax^®^manufactured by E.I.P.I.C.O., Egypt) every 12 h for one day.

### Surgical phase

**Fig. 2:**
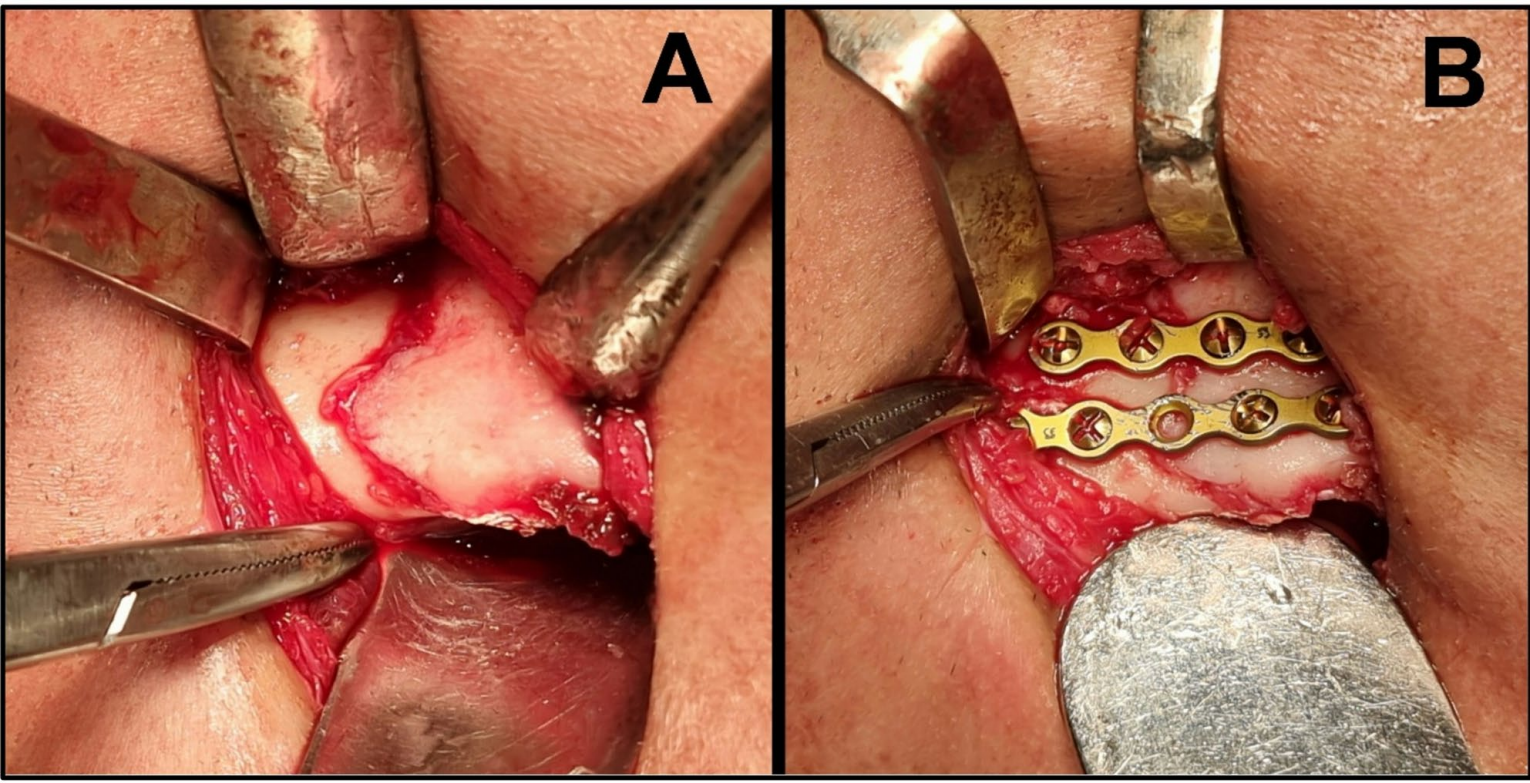
**Open reduction and internal fixation. A)** Extraoral access to the mandibular region via a submandibular (Risdon) incision placed below the inferior border of the mandible. **B)** Double 2 mm titanium miniplates securely adapted and fixed across the fracture line. The miniplates are positioned parallel to each other, typically along the inferior border of the mandible and anchored with bi-cortical 2 mm diameter screws to ensure stable fixation.

**Fig. 3:**
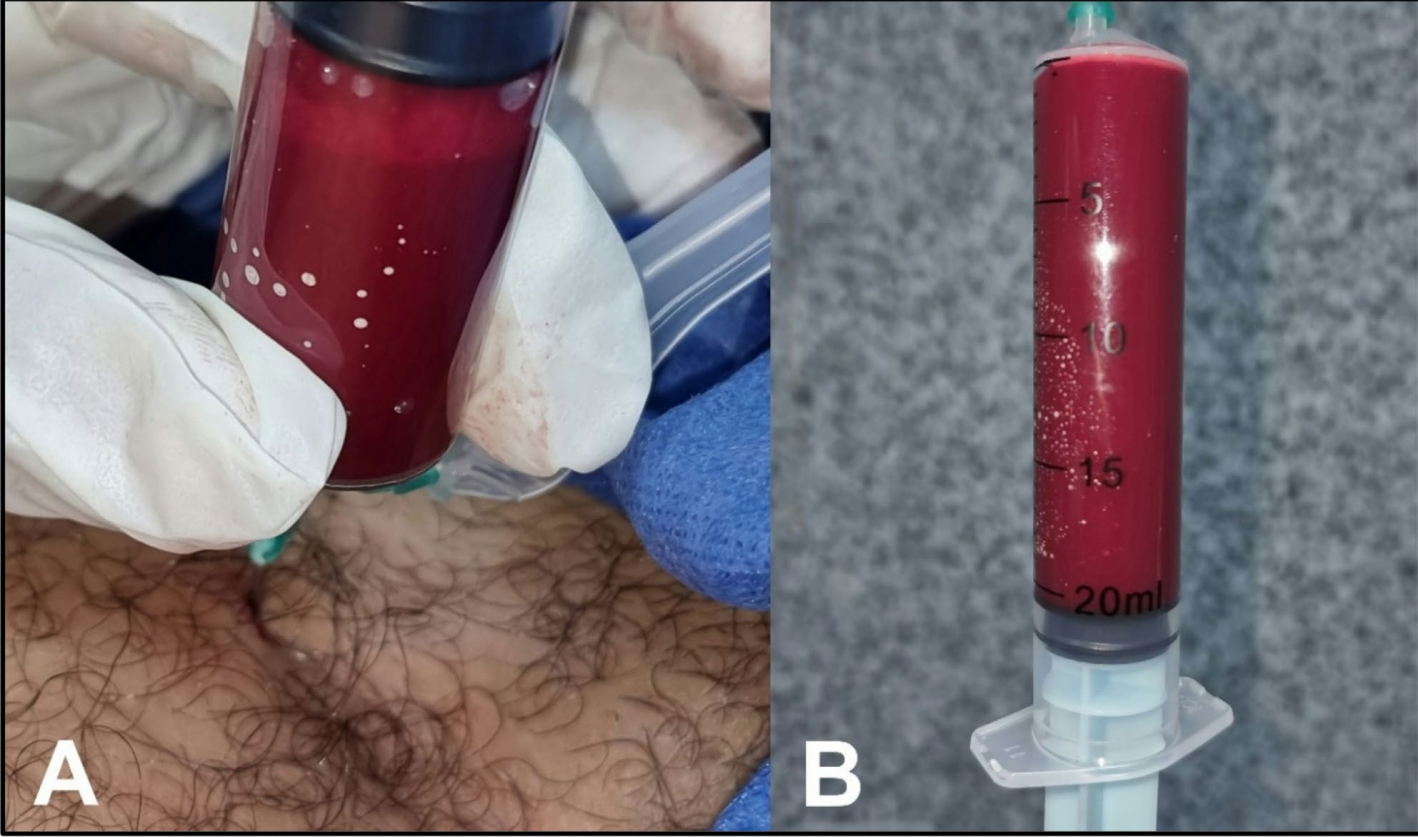
**Blood collection procedure. A)** Aspiration of 20 cm arterial blood from femoral artery. **B)** Sterile syringe used to aspirate full of 20 cm^3^of blood.

**Fig. 4:**
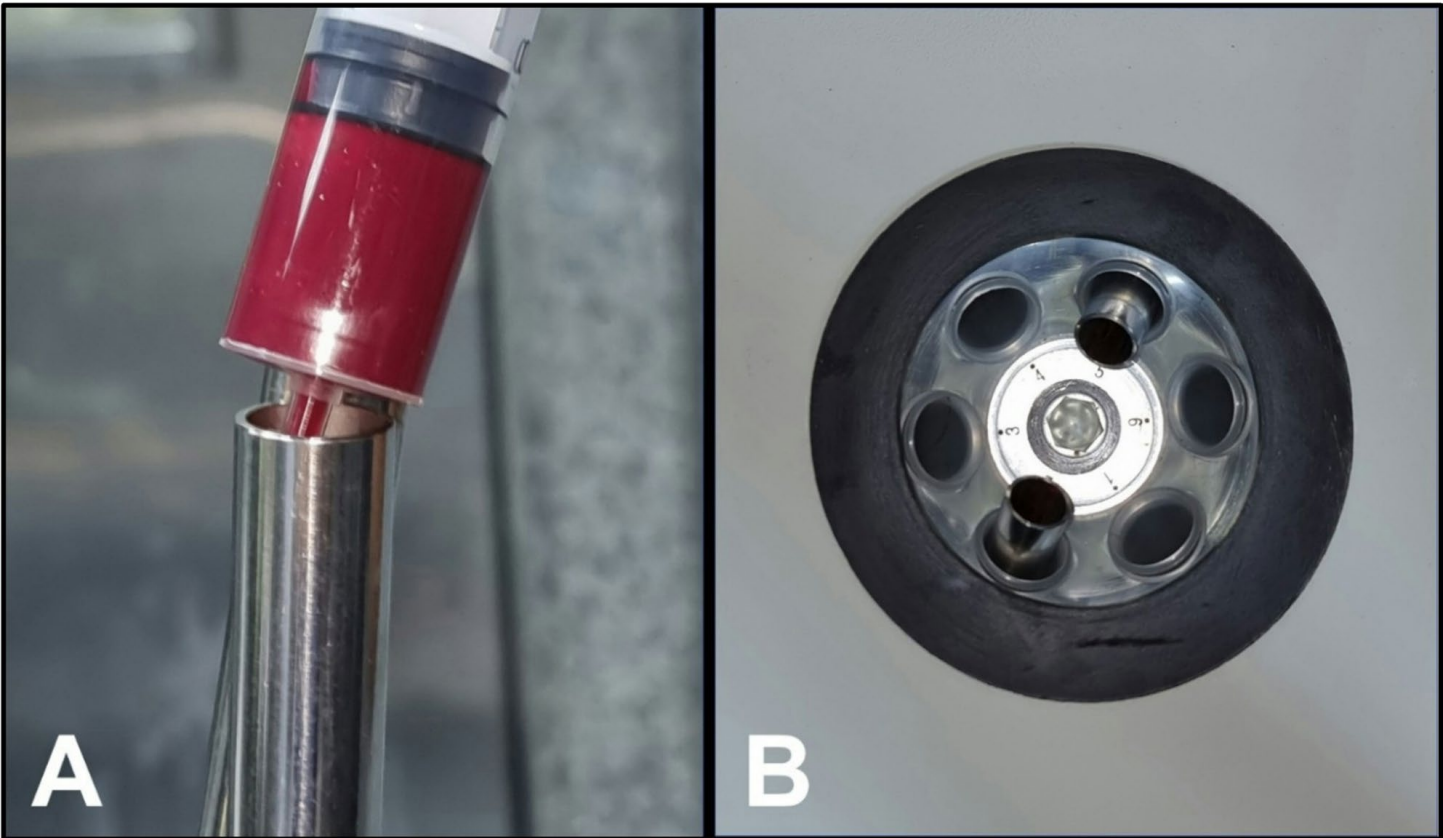
**T-PRF preparation. A)** Blood was transferred into sterile, grade IV titanium tubes without the addition of anticoagulants. **B)** Tubes are centrifuged at 2800 rpm for 12 minutes at room temperature.

**Fig. 5:**
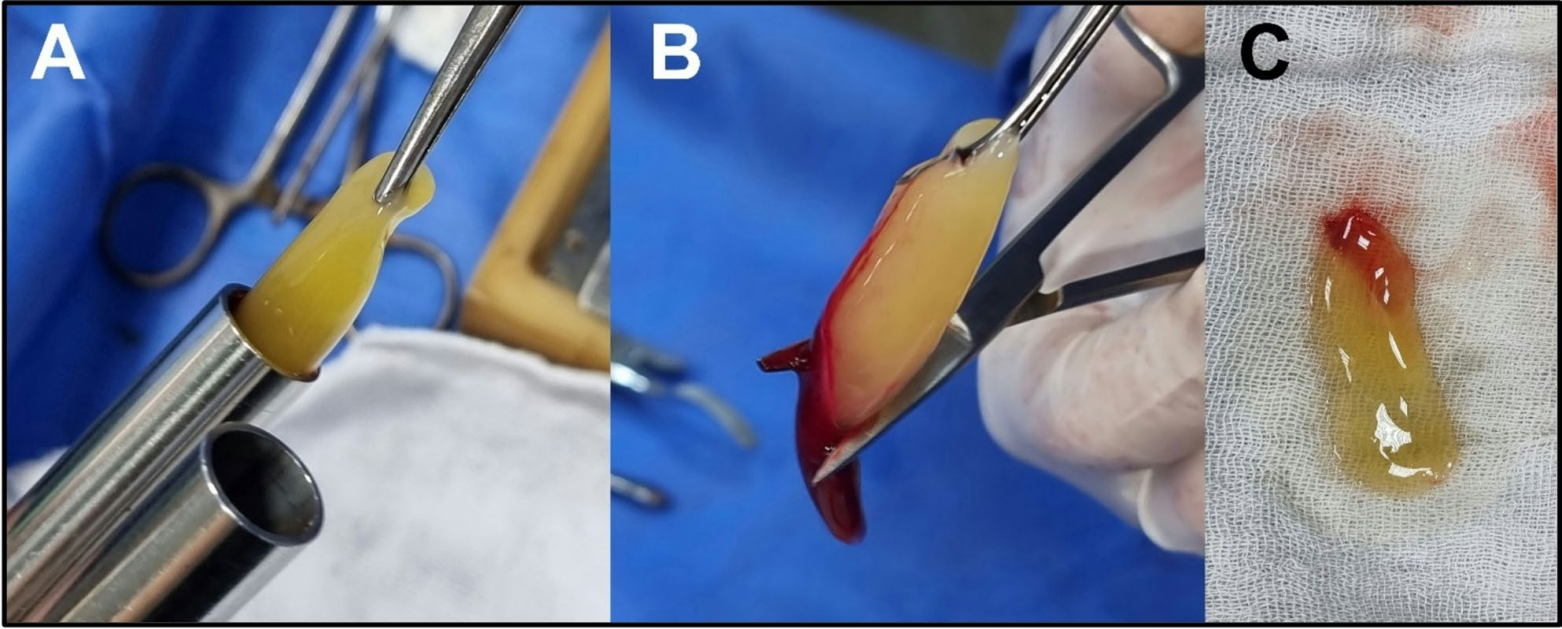
**Processing of freshly prepared T-PRF. A)** Withdrawal of T-PRF using sterile tissue forceps. **B)** Separation of blood clot from the distal end of T-PRF.**C) **T-PRF placed on sterile gauze and allowed to rest for 20 minutes.

**Fig. 6:**
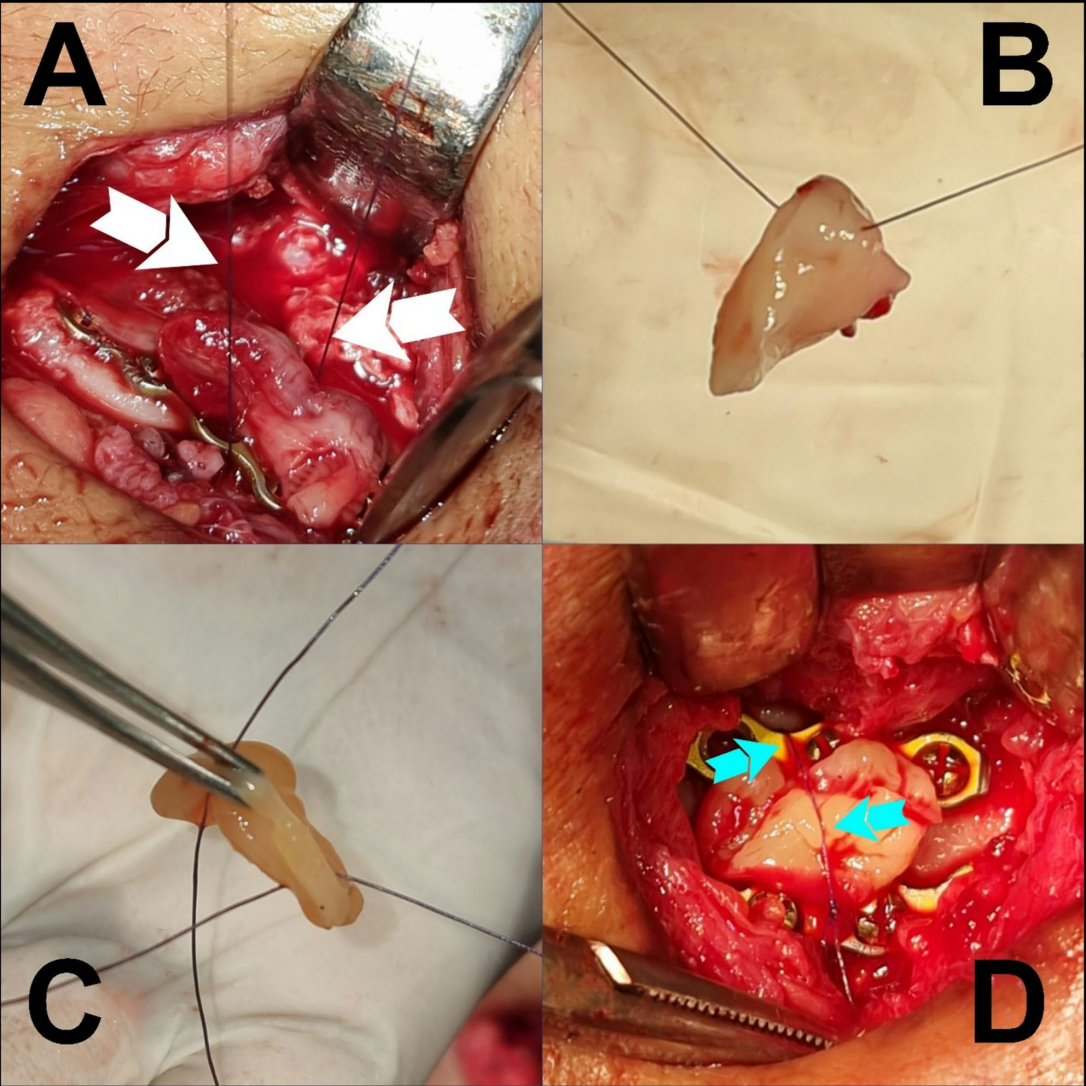
**Fixation of T-PRF in place. A)** Carefully threading 3/0 vicryl sutures beneath both miniplates. White arrows indicate both suture’s ends after crossing under the plates. **B **and** C)** Passing the stay suture through T-PRF. **D)** Positioning T-PRF over the fracture site then looping stay sutures around the T-PRF, effectively circumscribing and securing the membrane in direct contact with the underlying bone. Blue arrows points at the suture’s holding the T-PRF in place.

**Fig. 7:**
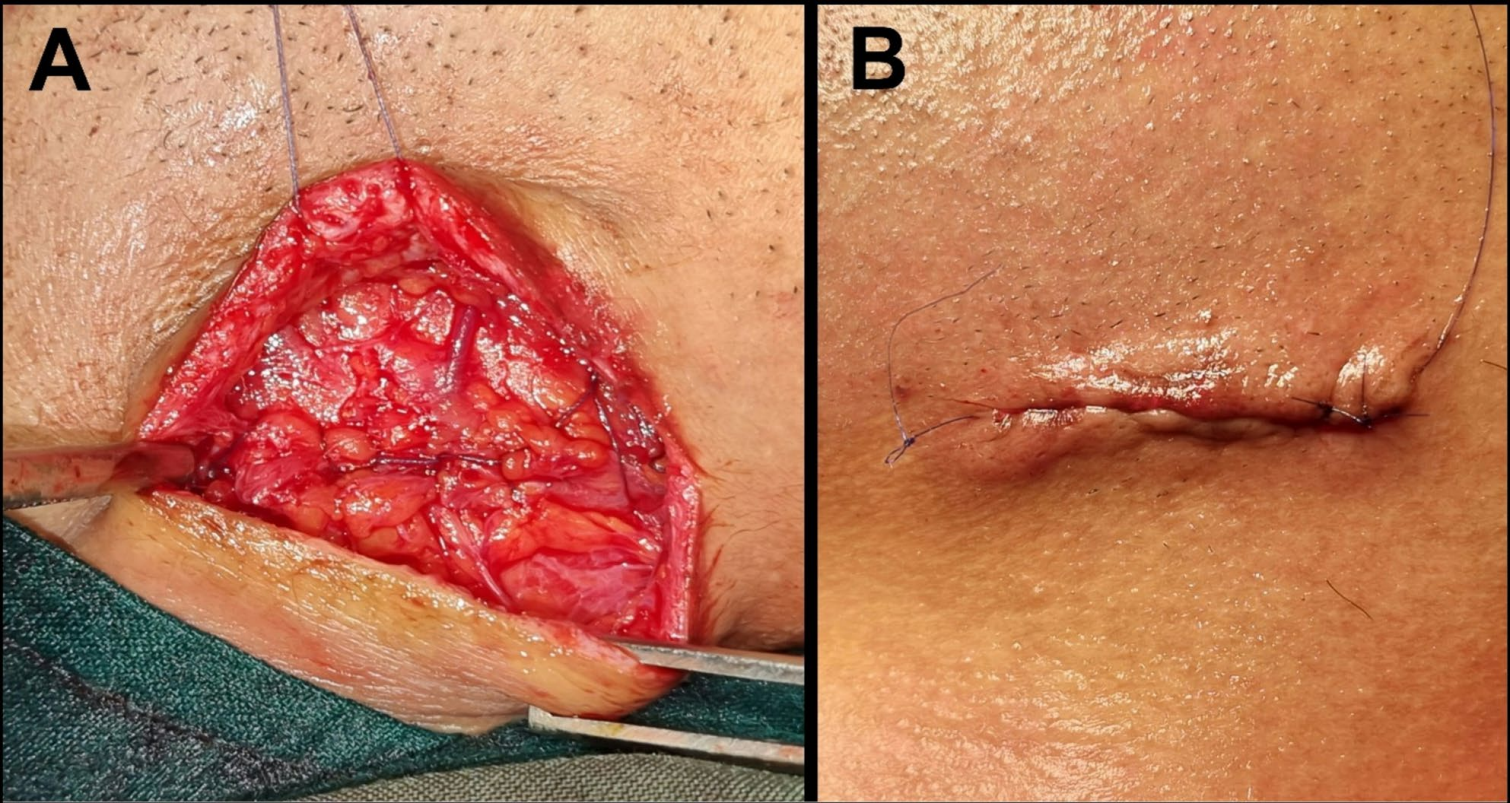
**Layered closure of a surgical Risdon (submandibular) incision. A)** Reapproximating of 1^st^layer (pterygomasseteric sling and platysma muscle) using resorbable 3/0 vicryl sutures. **B)** Skin layer closure by running subcuticular suture using non-resorbable 5/0 monofilament polypropylene.

1. All patients underwent general anesthesia with nasotracheal intubation.
2. The surgical site was prepared using a povidone-iodine scrub solution, after which the patient was draped with sterile towels, exposing only the operative field.
3. Intermaxillary fixation (IMF) was achieved by applying Erich arch bars with circum-dental stainless-steel wires or Interdental eyelet wiring (Ivy loop method). The occlusion was then placed into maxillomandibular fixation using stainless steel wire loops [20].
4. Surgical exposure of the fracture(s) was accomplished either intraorally via a vestibular approach using a Bard-Parker blade number 15, or extraorally via a Risdon incision, which was made as a skin incision using a Bard-Parker blade number 10 approximately 2 cm below the angle or inferior border of the mandible, measuring 4–5 cm in length. After the platysma muscle was cut, bony fragments were reached by blunt dissection (Fig. 2A). The choice of incision was determined by the anatomical location of the fracture [4].
5. Following surgical exposure, the fracture line was mobilized using a chisel or reduction forceps to facilitate anatomical reduction. Any soft tissue interposed within the fracture gap was meticulously removed with a surgical curette or the sharp edge of a periosteal elevator.
6. Teeth within the fracture line were either extracted or preserved based on clinical assessment of their viability and potential to impede healing.
7. Standard 2.0 mm system titanium miniplates were anatomically contoured using bending pliers. The first plate was positioned along the inferior mandibular border.
8. Using a plate-compatible drill, bi-cortical holes were placed through the plate**—** avoiding tooth roots and neurovascular structures**—** with at least two holes on either side of the fracture line.
9. Titanium screws measuring 2 mm in diameter and 9–13 mm in length were inserted bi-cortically to secure the fracture fixation plate, with two screws placed per fracture side using the corresponding screwdriver. The screw length was selected according to mandibular cortical thickness.
10. A second plate was then positioned and secured by screws superior to the first plate, maintaining an inter-plate distance of 3–5 mm (Fig. 2B).
11. Reduction accuracy was confirmed by ensuring lingual cortex continuity and flush alignment of mandibular borders. Maxillomandibular fixation was subsequently released, and occlusal harmony was verified.

12. T-PRF preparation and placement [21, 22]
  Two grade IV custom-made titanium tubes (Arab Engineers Co, Asyut, Egypt) were employed for T-PRF preparation. A single venipuncture was performed on either the right or left arm/leg to collect 20 mL of whole blood (Fig. 3), with 10 mL transferred into each tube (Fig. 4A). The blood was rapidly collected and centrifuged at 2,800 rpm for 12 min at room temperature (Fig. 4B). Following centrifugation, T-PRF clots were aseptically extracted using sterile tweezers (Fig. 5A), meticulously separated from the red blood cell layer (while retaining a thin red blood cell layer) with surgical scissors (Fig. 5B), and placed on sterile gauze. The T-PRF was allowed to rest for 20 min to facilitate gradual serum release (Fig. 5C). T-PRF was longitudinally bisected to ensure adequate fracture line coverage, positioned along the fracture site, and secured with Vicryl 3/0 resorbable sutures (Fig. 6).

13. The incision was closed in layers using 3/0 resorbable Vicryl sutures, with either simple interrupted or continuous locking sutures to approximate the first tissue layers (pterygomasseteric sling and platysma muscle). The epidermal layer was closed with a running subcuticular suture using 5/0 monofilament polypropylene, which was removed 5 days later (Fig. 7).

### Post-operative phase

#### Early postoperative care

In the immediate postoperative period, all patients were instructed to apply ice packs externally to the surgical site for the first 24 h, followed by warm compresses until edema resolution [23].

#### Postoperative medication

All patients received the following standardized postoperative medication regimen for pain and edema control.

- Antibiotic prophylaxis included intravenous cefotaxime (1 gram every 12 h) on the first postoperative day, transitioning to oral amoxicillin/clavulanate (1 gram, Augmentin^®^, GlaxoSmithKline, UK) **—** or oral clarithromycin (Klacid 500^®^, Abbot Laboratories, USA) if sensitivity occurred **—** twice daily for five days.
- Pain management comprised oral diclofenac potassium (50 mg, Cataflam^®^, Novartis, Switzerland) **—** a nonsteroidal anti-inflammatory drug (NSAID) **—** administered every eight hours for five days.
- Metronidazole (Flagyl^®^: metronidazole 500 mg by GlaxoSmithkline, UK) 500 mg every8 hours for 5 days.
- Alpha-chemotrypsin (Alpha-chemo-trypsin^®^: Leurquin France, packed by Amoun Pharmaceutical CO.S.A.E., Egypt) ampules once daily for 5 days.
- Patients were prescribed chlorhexidine-based antiseptic mouthwash for oral hygiene. Dietary guidelines mandated a soft, liquid diet rich in high-protein and high-calorie intake for four weeks post-surgery.
- Oral hygiene was maintained in all patients using conventional toothbrushing and warm saline rinses.

### Laser biomodulation in study group

**Fig. 8:**
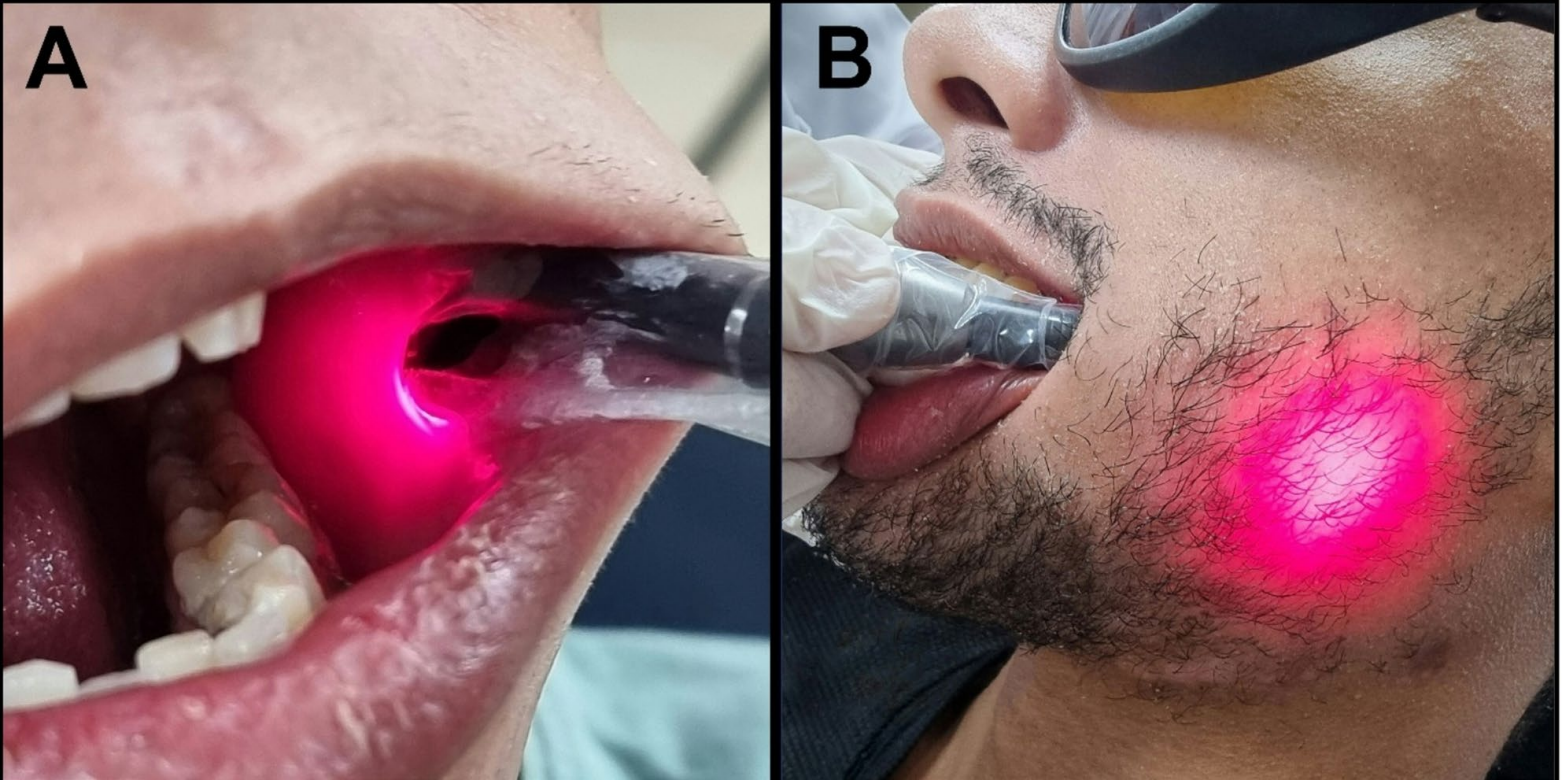
**Intraoral application of LLLT stimulation. A)** Precise positioning of the laser handpiece directly over the area of interest within the oral cavity (posterior to the third molar). Holding the device perpendicular to the mucosal surface above the surgical site. **B)** Laser beam application intraorally at surgical site with its emission penetrating the cheek tissues and becoming visible externally. This figure illustrates the ability of specific laser wavelengths, particularly in the near-infrared range, to traverse multiple soft tissue layers, allowing for both intraoral and extraoral therapeutic applications.

Subjects related to group B (Study group) underwent the process of biostimulation of the fracture line using low-level laser therapy (Fig. 8). A diode laser device (SIROLASE BLUE™, DENTSPLY SIRONA, USA) was used and adjusted at the following settings [24]:

- Wavelength [λ]: 660 nm (red wavelength).

- Power: 100 mW. 

- Exposure time was continuous mode and for 60 seconds.

**Fig. 9:**
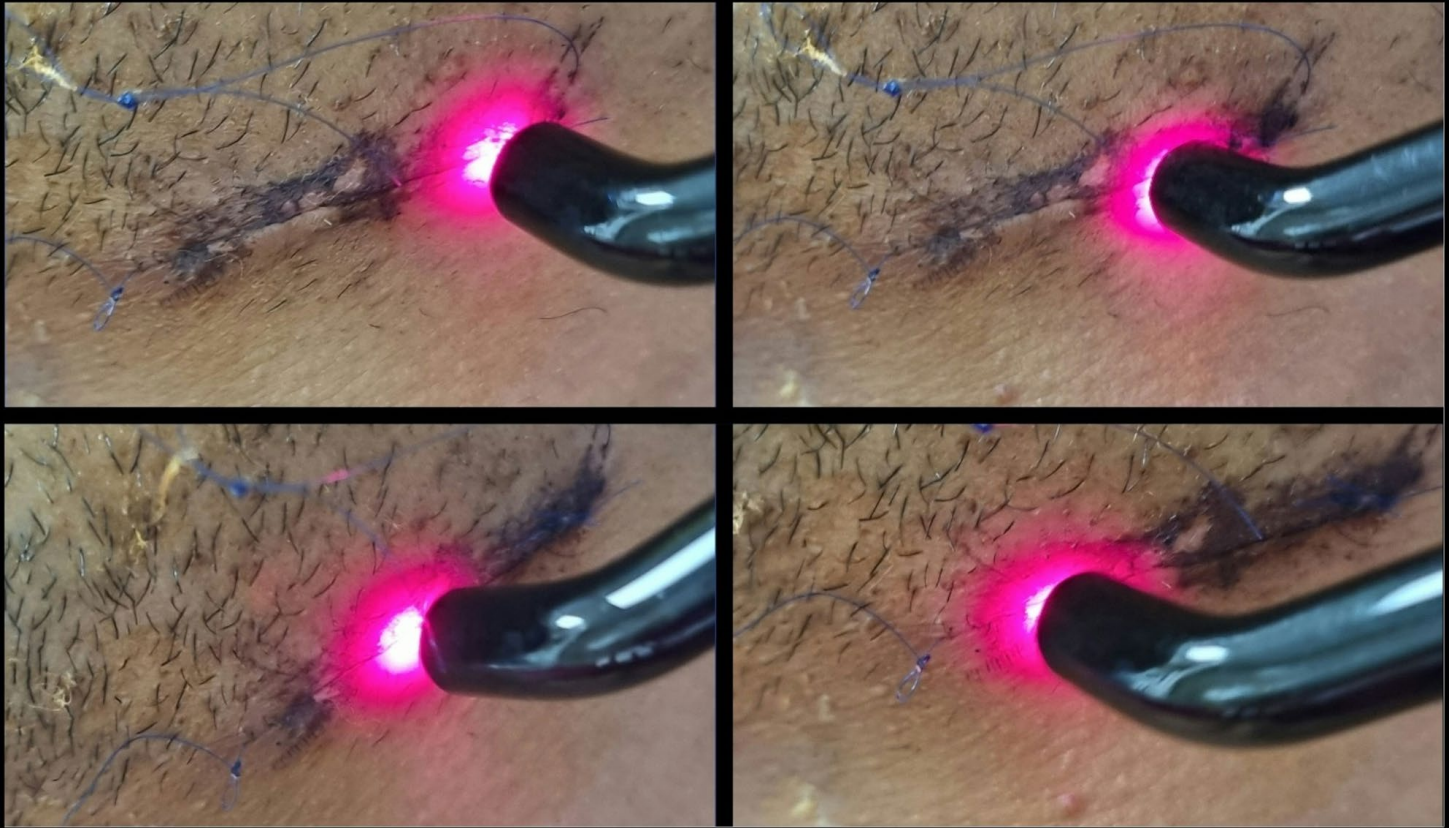
**Extra-oral application of LLLT using a scanning motion over the surgical incision. **Laser handpiece positioned externally on the skin surface, directly above the underlying surgical site moving in a controlled, continuous scanning motion along the length of the incision to ensure uniform photobiomodulation across the entire area

Treatment was administered using an 8 mm MultiTip applicator, delivering a fluence of approximately 11.9 J/cm² per pulse. Pulses were pointed along the incision line at a minimum of three points (Fig. 9); each point received three complete pulses. Each patient was subjected to 4 sessions on 1 st day, 1 st week, 2nd week, and 3rd week postoperatively.

### Parameters for evaluation

#### Clinical evaluations

Conducted at standardized postoperative intervals: 24 hours, 1 st week, 2nd week, 3rd week, 4 weeks, 6 weeks, and 12 weeks.

i. Postoperative dental occlusion was assessed during the whole follow-up period to evaluate the establishment of proper inter-arch relationships, including Angle’s molar and canine classifications and maxillomandibular dental midline alignment. Occlusal discrepancies, such as anterior open bite or premature posterior contacts, were systematically recorded.
ii. Maximal Mouth Opening (MMO) was quantified at 24 hours, 1 st week, 4 weeks, 6 weeks, and 12 weeks. Intraoral method – the gold standard method – was implemented by using sterilizable metallic ruler to measure the distance between the incisal edges of the upper and lower anterior teeth in millimeters (mm) to evaluate tissue repair progression, masticatory muscle tone recovery, and restoration of physiological mandibular movement [25].
iii. Postoperative edema was graded at 24 hours, 1 st week, 2nd week, and 3rd week based on pitting depth (visually measured) and recovery time using a standardized scoring system, as follows: Grade 0: No clinical edema; Grade 1: Slight pitting (2 mm depth), rebounds immediately; Grade 2: Somewhat deeper pit (4 mm), fewer than 15 seconds to rebound [26].
iv. Pain intensity was tracked using a 10-point Visual Analogue Scale (VAS); 0 means no pain and 10 means worst pain ever, at the standardized postoperative intervals of 24 hours, 1 st week, 2nd week, and 4 weeks to analyze post-operative pain progression during healing [27].
v. Wound healing progression was systematically assessed at 24 hours, 1 st week, 2nd week, and 3rd week using the Early Wound Healing Score (EHS), which evaluates clinical parameters independently recorded and analyzed as follows [28]:
  1. Clinical signs of re-epithelization (CSR); 0 points: visible distance between incision margins; 3 points: contact between incision margins; 6 points: merged incision margins.
  2. Clinical signs of hemostasis (CSH); 0 points: bleeding at the incision margins; 1 point: presence of fibrin on the incision margins; 2 points: absence of fibrin on the incision margins.
  3. Clinical signs of inflammation (CSI); 0 points: redness involving >50% of the incision length and/or pronounced swelling; 1 point: redness involving <50% of the incision length; 2 points: absence of redness along the incision length.

#### Radiographic evaluation

**Fig. 10:**
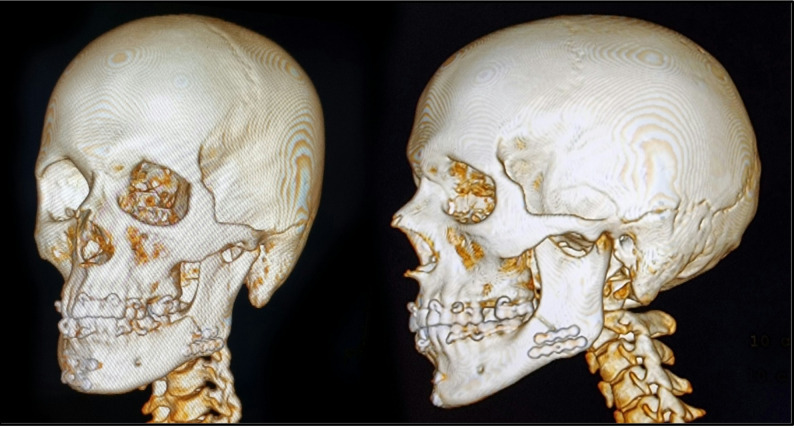
**3D window of Postoperative CT view. **CT images demonstrate adequate reduction and fixation of the fracture and precise anatomical alignment of the bone fragments with restoration of normal contour and continuity.

**Fig. 11:**
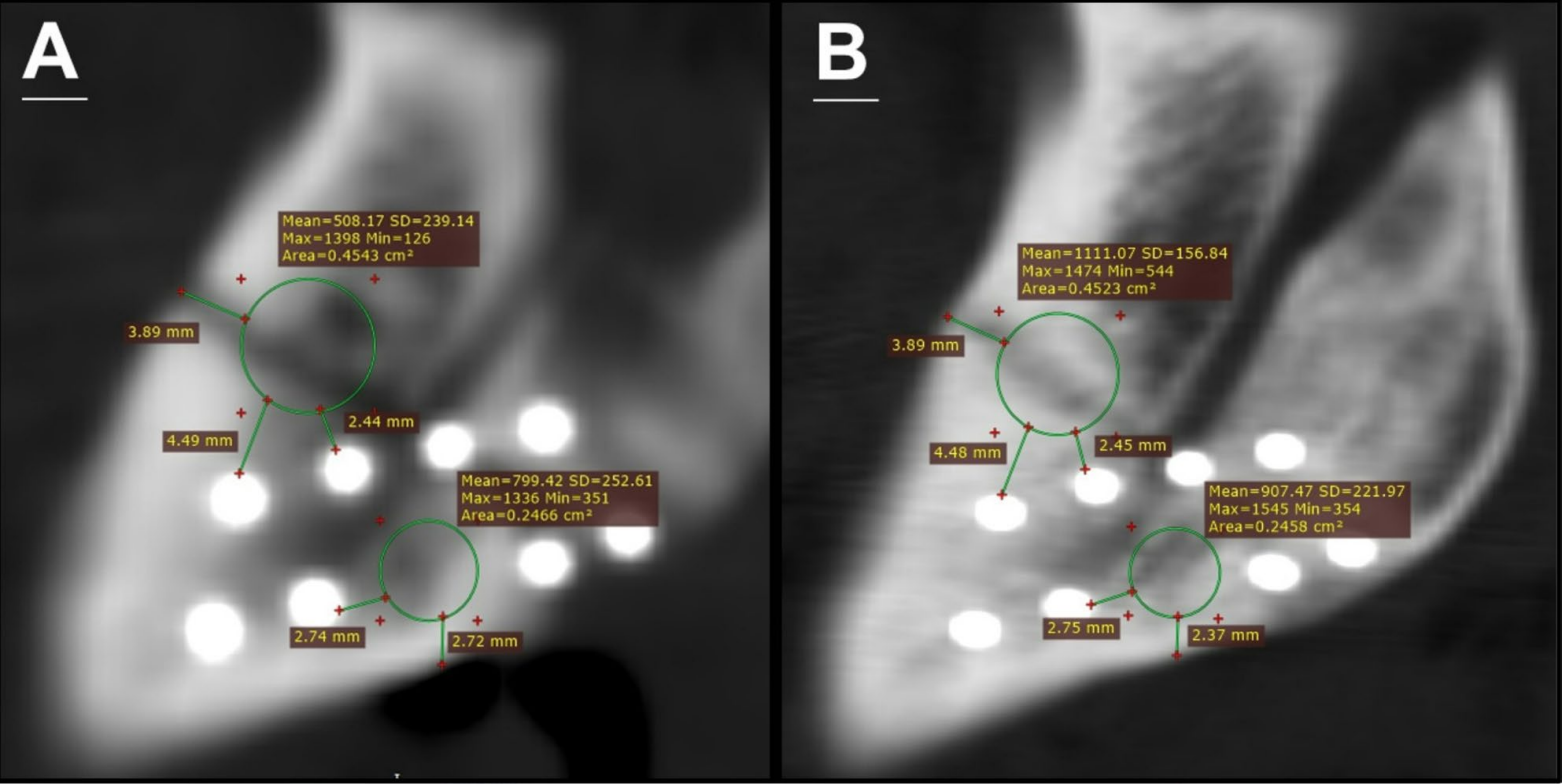
**Sagittal view, CT scan analysis of bone density in HU using RadiAnt® DICOM Viewer software. A)** Immediate post-operative CT scan analysis of bone density in HU at region of interest delineated over the fracture site. **B)** 12 weeks follow-up CT scan analysis of bone density in HU at region of interest delineated over the fracture site

**Fig. 12:**
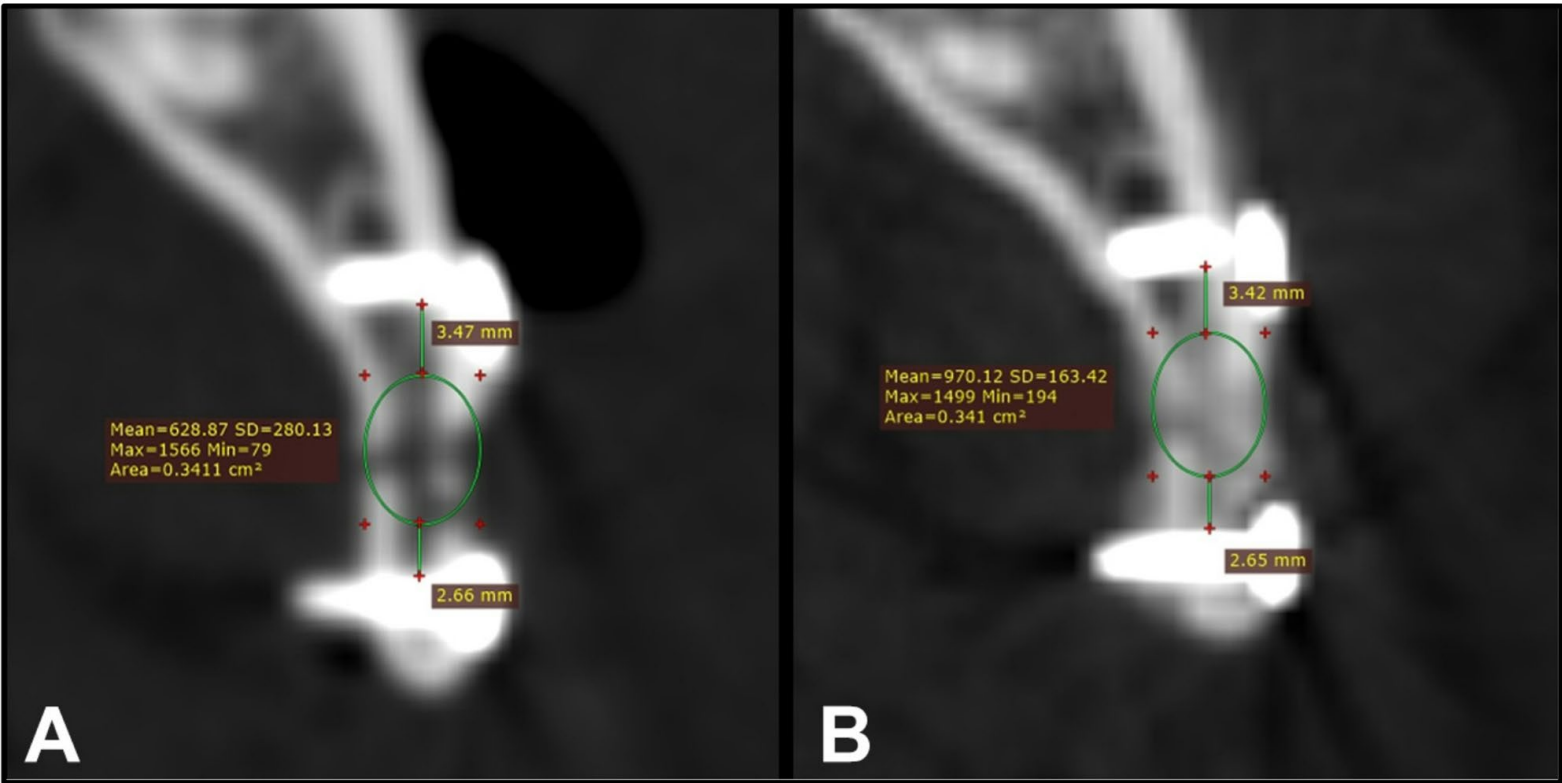
**Axial view, CT scan analysis of bone density in HU using RadiAnt® DICOM Viewer software. A)** Immediate post-operative CT scan analysis of bone density in HU at region of interest delineated over the fracture site. **B)** 12 weeks follow-up CT scan analysis of bone density in HU at region of interest delineated over the fracture site

i. An immediate postoperative computed tomography (CT) scan was performed to assess fracture reduction accuracy, fixation stability, and baseline bone mineral density in Hounsfield Units (HU) (Fig. 10).
ii. A follow-up CT scan was conducted at 12 weeks (3 months) to quantify bone mineral density changes relative to baseline. Axial, coronal, and sagittal reconstructions were analyzed using RadiAnt® DICOM Viewer software (Version 2025.1.2, Medixant) to calculate HU values. A consistent region of interest was defined across both scans using an elliptical tool to measure mean bone density (± standard deviation) within the fracture site (Fig. 11, 12).

### Statistical analysis^4^

Data was analyzed using IBM SPSS, version 23 for Windows, Armonk, NY, USA. Data normality was checked using Shapiro Wilk test and Q-Q plots. All variables revealed a non-normal distribution; thus, median and inter-quartile range (IQR) were mainly used for data presentation in addition to mean and standard deviation (SD). Comparison between groups was done using Mann Whitney U test, while within group differences were analyzed using Wilcoxon Signed Rank test or Friedman test, which was followed by pairwise comparisons with Bonferroni correction. Gender was analyzed using Chi-Square test. All tests were two-tailed, and the significance level was set at p value < 0.05.

## Results

### Demographic and clinical characteristics

**Fig. 13:**
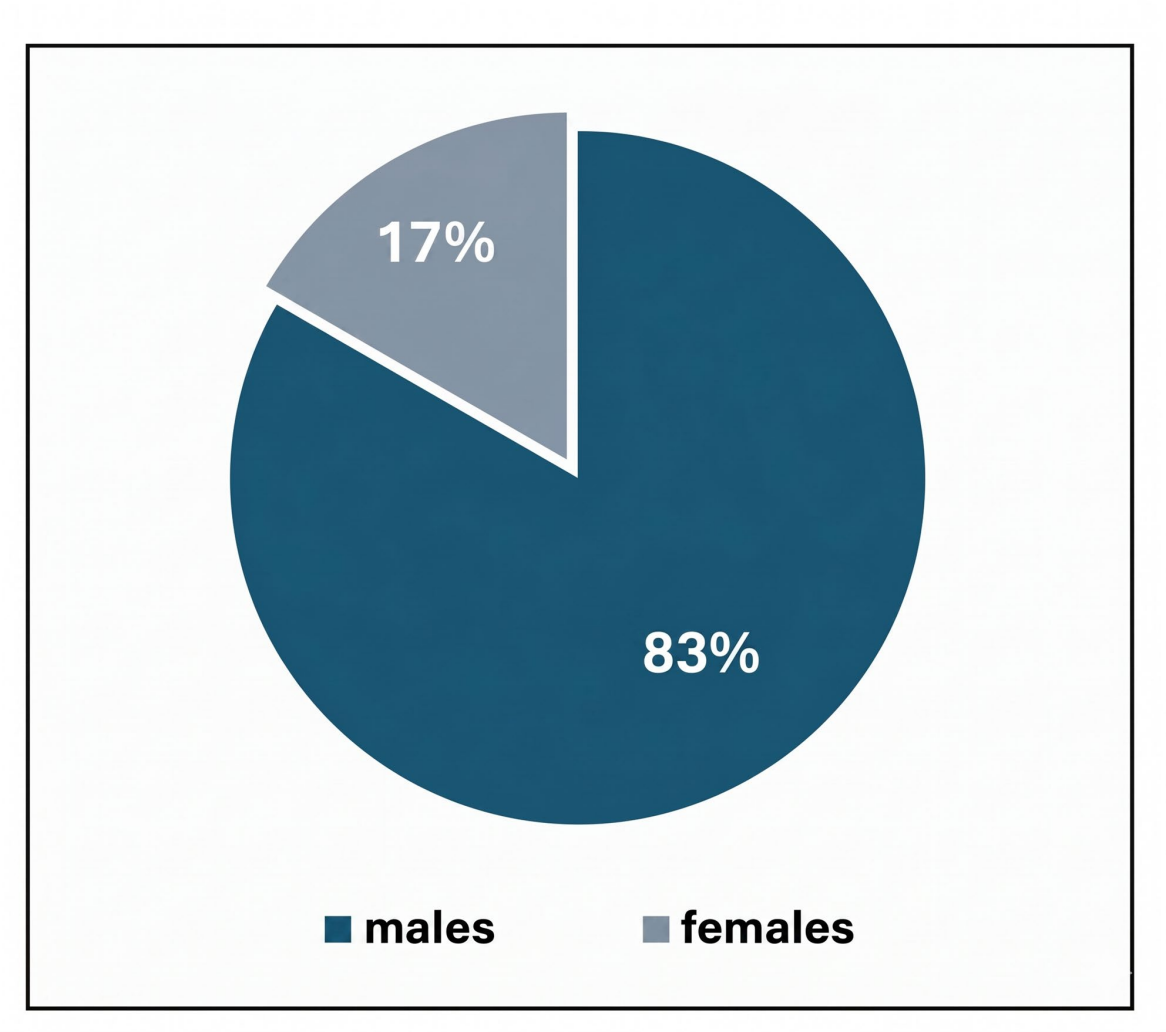
**Proportion of males to females as a percentage of the total sample size. **Chart illustrating the male-to-female ratio within the study population (5 males: 1 female)

A total of 12 patients with 14 mandibular fracture lines were included in the study, comprising 9 males and 3 females (male-to-female ratio: 3:1) (Fig. 13). Patients were evenly allocated to two groups (*n* = 6 per group). The age range in the control group was 20–40 years with a mean of 32.50 ± 12.75 years, and in the study group was 21–39 years with a mean of 29.50 ± 9.99 years (Table 1). The primary etiology of mandibular fractures was road traffic accidents (RTAs) (58%, *n* = 7), followed by falls (25%, *n* = 3), direct trauma (8.5%, *n* = 1), and gunshot wounds (8.5%, *n* = 1).

**Table 1:** Demographic data of the study participants

		Control	Study	*P* value
Age in year	Mean ± SD	32.50 ± 12.75	29.50 ± 9.99	0.845
	Median (IQR)	29.50 (21.25)	24.50 (18.00)	
Gender: n (%)	Males	5 (83.33%)	4 (66.7%)	0.347
	Females	1 (16.67%)	2 (33.3%)	
Mode of trauma: n (%)	Falls	2 (50%)	1 (16.7%)	0.429
	RTA	3 (37.5%)	4 (66.7%)	
	Direct trauma	0 (0%)	1 (16.7%)	
	Gun shot	1 (12.5%)	0 (0%)	

### Preoperative findings

All patients presented with facial asymmetry due to swelling and tenderness at the fracture site. Malocclusion was observed in 11 patients. Seven patients had a tooth in the fracture line, four exhibited mouth deviation toward the affected side, and three cases reported inferior alveolar or mental nerve paresthesia before surgery. A total of nine patients were admitted with bilateral mandibular fractures: five were related to the study group and four to the control group (regardless of fracture place and its inclusion or exclusion from the final analysis).

All patients underwent open reduction and internal fixation using conventional double miniplates. The mean preoperative hospitalization period for edema resolution was 5 days.

### Postoperative assessment

Postoperative clinical evaluations were conducted at standardized postoperative intervals: 24 hours, 1 st week, 2nd week, 3rd week, 4 weeks, 6 weeks, and 12 weeks according to each related parameter.

### Clinical outcomes

#### Occlusal and bony relationships

Occlusal examination demonstrated normal midline alignment, occlusal and normal inter-cuspal relationships in all cases, with no need for selective grinding or occlusal adjustment.

#### Maximum mouth opening

**Fig. 14:**
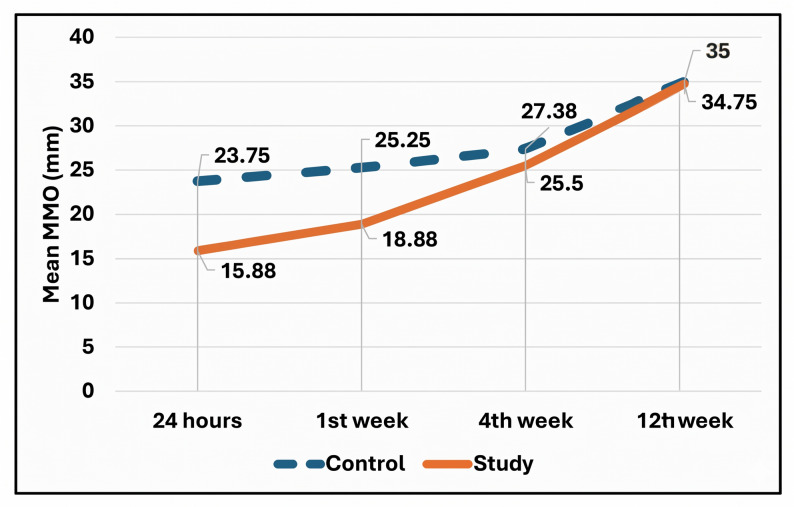
**Comparison between 24 hours, 1 week, 4 weeks and 12 weeks postoperative according to Maximum Mouth Opening (MMO).**

The maximum mouth opening was assessed at regular intervals of 24 hours, 1 st week, 4 weeks, and 12 weeks. At 24 hours postoperatively, the study group had a mean MMO of 15.88 ± 2.53 mm, while the control group averaged 23.75 ± 6.85 mm. By the 12-week follow-up, all patients had regained normal mouth opening (study group: 34.75 ± 7.80 mm; control group: 35.00 ± 4.76 mm) (Fig. 14). No statistically significant difference in MMO was observed between groups at any time point (Table 2).

**Table 2:** Comparison of mouth opening (mm) between study groups

MMO/Group		Control	Study	*P* value^1^	Effect size
24 h	Mean ± SD	23.75 ± 6.85	15.88 ± 2.53	0.081	1.524
	95% CI	12.85, 34.65	11.85, 19.90		
	Median (IQR)	20.50 (10.80)	15.75 (4.90)		
1 st week	Mean ± SD	25.25 ± 7.19	18.88 ± 3.71	0.327	1.113
	95% CI	13.80, 36.70	12.98, 24.77		
	Median (IQR)	22.00 (11.50)	18.50 (7.10)		
4th week	Mean ± SD	27.38 ± 8.50	25.50 ± 6.46	0.831	0.249
	95% CI	13.85, 40.90	15.23, 35.77		
	Median (IQR)	26.75 (16.40)	24.00 (12.00)		
12th week	Mean ± SD	35.00 ± 4.76	34.75 ± 7.80	0.567	0.039
	95% CI	27.42, 42.57	22.33, 47.17		
	Median (IQR)	35.00 (9.00)	35.00 (14.80)		
*P* value^2^		0.026*	0.011*		

*Statistically significant difference at p value < 0.05

*P* value^1^: Mann Whitney U test

*P* value^2^: Friedman test

Only one patient in the study group exhibited persistent trismus for two weeks postoperatively, with minimal improvement in MMO. Management consisted of muscle relaxants and regular stretching exercises using tongue blades. By the fourth postoperative week, this patient demonstrated MMO values comparable to those observed in the other cases within the study group.

#### Postoperative edema

**Fig. 15:**
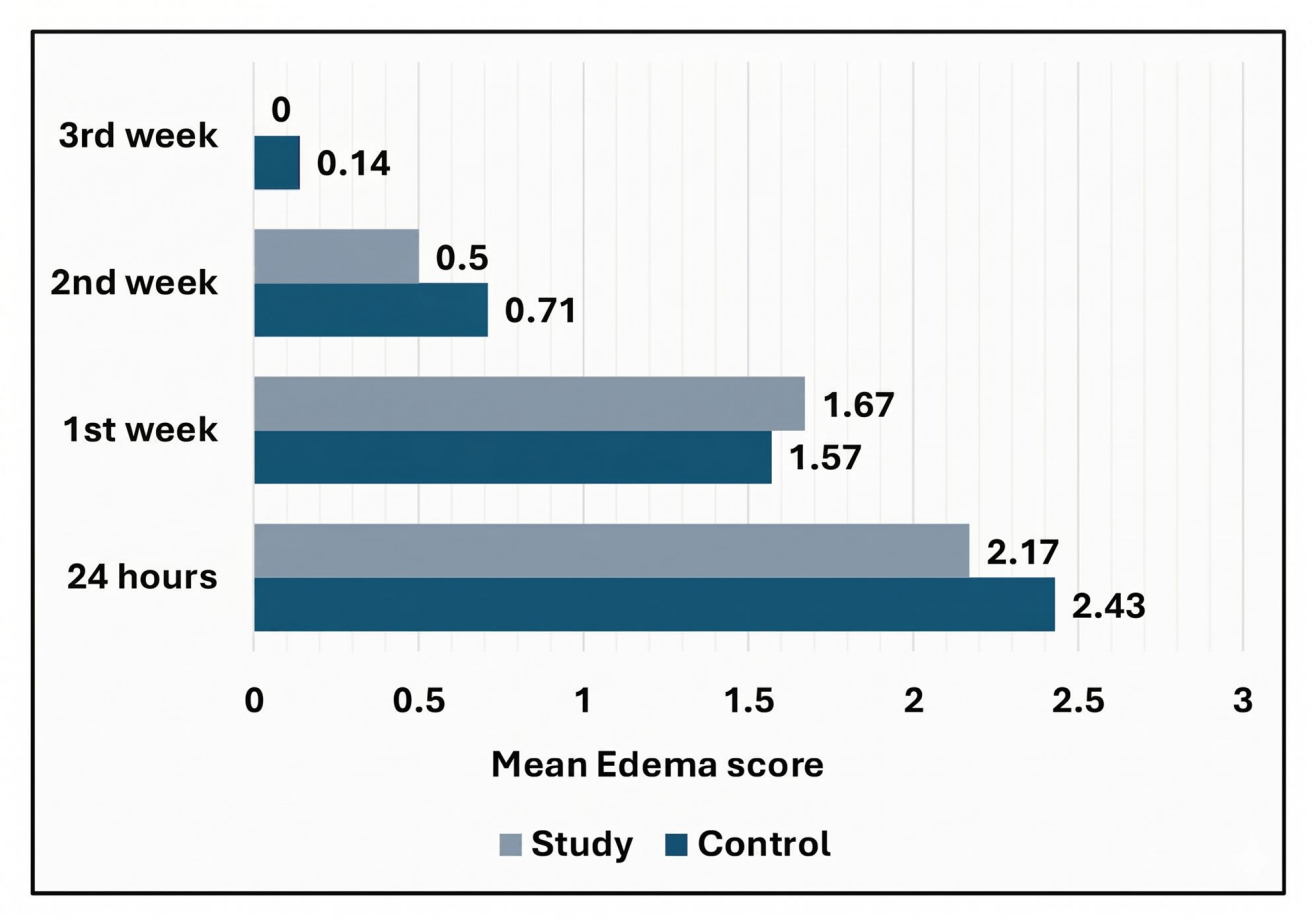
**Comparison between 24 hours, 1 week, 2 weeks and 3 weeks postoperative according to Edema score.**

Pitting depth was monitored at 24 hours, 1 st week, 2nd week, and 3rd week showed that edema resolved in most cases by days 7–10, with only one case in the control group persisting for 18 days. By week two, 91% of patients had minimal or no edema, and all were free of swelling by week three. A statistically significant reduction in edema was observed over time in each group (*p* ≤ 0.05), with no significant difference between groups at any time point (Table 3; Fig. 15). Periodontal tissues remained healthy throughout the follow-up, with no evidence of gingival pathology or inflammation postoperatively.

**Table 3:** Comparison of edema scores between study groups

Edema score/Group		Control	Study	*P* value^1^	Effect size
24 h	Mean ± SD	2.43 ± 0.53	2.17 ± 0.75	0.521	0.400
	95% CI	1.93, 2.92	1.38, 2.96		
	Median (IQR)	2.00 (1.00)	2.00 (1.30)		
1 st week	Mean ± SD	1.57 ± 0.53	1.67 ± 0.52	0.735	0.190
	95% CI	1.07, 2.07	1.13, 2.21		
	Median (IQR)	2.00 (1.00)	2.00 (1.00)		
2nd week	Mean ± SD	0.71 ± 0.76	0.50 ± 0.55	0.633	0.317
	95% CI	0.02, 1.41	0.00, 1.08		
	Median (IQR)	1.00 (1.00)	0.50 (1.00)		
3rd week	Mean ± SD	0.14 ± 0.38	0.00 ± 0.00	0.355	0.521
	95% CI	0.00, 0.49	0.00, 0.00		
	Median (IQR)	0.00 (0.00)	0.00 (0.00)		
*P* value^2^		< 0.001*	0.001*		

*Statistically significant difference at p value < 0.05

*P* value^1^: Mann Whitney U test

*P* value^2^: Friedman test

#### Postoperative pain

**Fig. 16:**
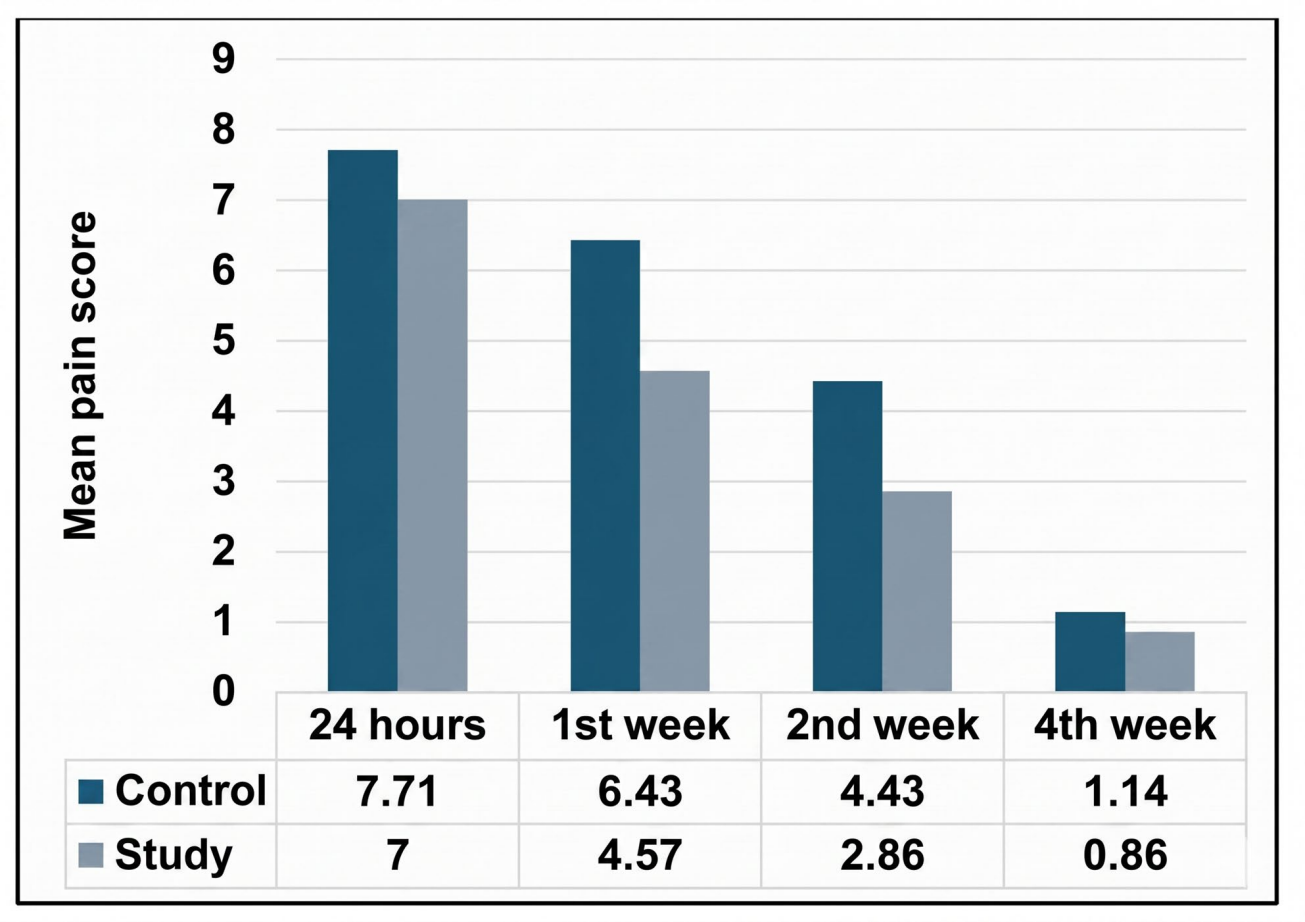
**Comparison between 24 hours, 1 week, 2 weeks and 4 weeks postoperative according to pain scores (visual analogue scale).**

Pain scores were assessed at 24 hours, 1 week, 2 weeks, and 4 weeks postoperatively. In the control group, pain scores decreased significantly from the second week onwards; similarly, also the study group exhibited significant improvement in pain scores starting from second week (*p* < 0.05). A statistically significant difference in pain reduction was observed between the two groups from the first to the second week (*p* < 0.05), with the study group demonstrating a greater reduction during this interval. However, at both the 24-hour and 4-week assessments, pain scores were comparable between the groups, and no statistically significant differences were detected at these time points (Table 4; Fig. 16). Persistent pain for more than ten days postoperatively was reported by five patients **—** three of whom were in the control group and the other two in the study group and were managed with additional analgesics and warm fomentation. Four patients experienced increased pain intensity in the second week compared to the first postoperative day, two of them were in the study group and had Methicillin-resistant Staphylococcus aureus (MRSA) infection, which was managed using microbial swabs for culture-guided antibiotic therapy. After MRSA was identified, an appropriate antibiotic protocol was prescribed (intravenous vancomycin for 5 days, followed by oral Tavacine 750 mgm -Hikma pharmaceutical, Egypt- for 9 days), along with wound irrigation and debridement. By the second week, all cases had resolved completely. Infection-control protocols for MRSA were carefully implemented, including the use of gowns, gloves, and, when appropriate, masks during all clinical interactions with MRSA-infected patients. Additionally, patients with confirmed MRSA infection were placed in isolated rooms, in accordance with established guidelines as described by Siddiqui and Koirala [29].

**Table 4:** Comparison of pain scores between study groups

Pain score/Group		Control	Study	*P* value^1^	Effect size
24 h	Mean ± SD	7.71 ± 1.38	7.00 ± 1.15	0.238	0.559
	95% CI	6.44, 8.99	5.93, 8.07		
	Median (IQR)	8.00 (2.00)	7.00 (2.00)		
1 st week	Mean ± SD	6.43 ± 1.62	4.57 ± 1.13	0.031*	1.331
	95% CI	4.93, 7.93	3.52, 5.62		
	Median (IQR)	7.00 (2.00)	4.00 (2.00)		
2nd week	Mean ± SD	4.43 ± 1.40	2.86 ± 0.38	0.009*	1.531
	95% CI	3.14, 5.72	2.51, 3.21		
	Median (IQR)	4.00 (2.00)2	3.00 (0.00)		
4th week	Mean ± SD	1.14 ± 1.46	0.86 ± 0.69	1.00	0.245
	95% CI	0.00, 2.50	0.22, 1.49		
	Median (IQR)	1.00 (2.00)	1.00 (1.00)		
*P* value^2^		< 0.001*	< 0.001*		

*Statistically significant difference at p value < 0.05

*P* value^1^: Mann Whitney U test

*P* value^2^: Friedman test

#### Wound healing

**Fig. 17:**
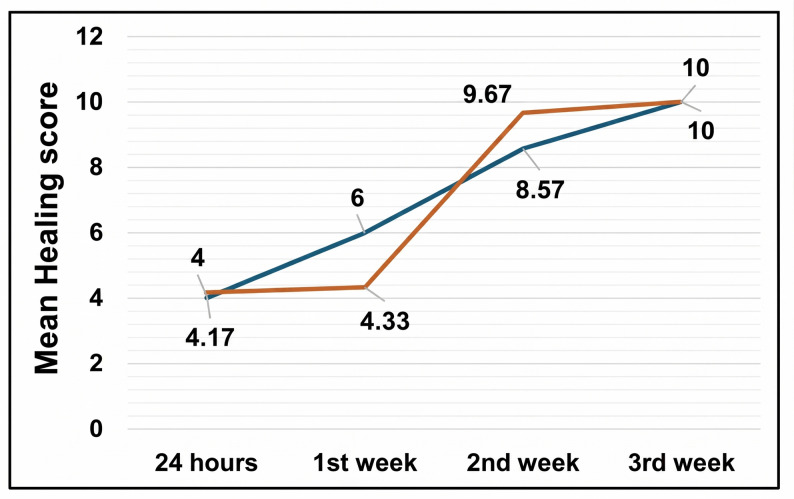
**Comparison between 24 hours, 1 week, 2 weeks and 3 weeks postoperative according to Wound Healing (Early wound healing score).**

Wound healing parameters were assessed at postoperative days 1, 7, 14, and 21. Each group showed significant improvement in healing parameters by week three. The control group exhibited significant improvement from week two (*p* < 0.05), while the study group showed marked acceleration by week three. Even though, no statistically significant difference was observed between the two groups in terms of all wound healing parameters (Table 5; Fig. 17). Also, epithelialization rates were comparable between groups, with no statistically significant difference at any time point.

**Table 5:** Comparison of healing scores between study groups

Healing score/Group		Control	Study	*P* value^1^	Effect size
24 h	Mean ± SD	4.00 ± 1.83	4.17 ± 0.75	0.535	0.12
	95% CI	2.31, 5.69	3.48, 4.86		
	Median (IQR)	5.00 (1.00)	4.00 (1.25)		
1 st week	Mean ± SD	6.00 ± 2.31	4.33 ± 3.39	0.472	0.58
	95% CI	3.86, 8.14	1.19, 7.47		
	Median (IQR)	5.00 (4.00)	5.00 (6.00)		
2nd week	Mean ± SD	8.57 ± 2.15	9.67 ± 2.94	1.00	0.43
	95% CI	6.58, 10.56	6.95, 12.39		
	Median (IQR)	10.00 (4.00)	9.00 (3.00)		
3rd week	Mean ± SD	10.00 ± 0.00	10.00 ± 0.00	1.00	0.00
	95% CI	10.00, 10.00	10.00, 10.00		
	Median (IQR)	10.00 (0.00)	10.00 (0.00)		
*P* value^2^		< 0.001*	0.001*		

*Statistically significant difference at p value < 0.05

*P* value^1^: Mann Whitney U test

*P* value^2^: Friedman test

Three patients in the study group experienced wound dehiscence within five days postoperatively. Two of these cases were associated with MRSA infections and were managed with antibiotics and surgical debridement, as previously described. The third case resulted from patient noncompliance with postoperative instructions and poor oral hygiene, leading to the separation of the wound edges; this was managed by wound refreshment, irrigation, debridement, and re-suturing with 3 − 0 silk sutures, which were removed after one week. All patients with wound dehiscence demonstrated clinical improvement within 7 to 10 days following intervention.

### Other complications

Of the three cases (two in the control group and one in the study group) presenting with preoperative nerve paresthesia, one patient- who was in the control group- experienced immediate resolution after surgery. In the remaining cases, management included vitamin B12 supplementation (Depovit B12, PHARCO PHARMACEUTICALS, Egypt) at a dose of 1000 mcg three times per week. The other control group patient showed recovery between the fourth and fifth postoperative weeks, while the patient in the study group recovered by the third week. No new sensory disturbances were reported in any case postoperatively.

### Radiographic findings (Table 6; Fig. 18)

**Table 6:** Comparison of bone density between study groups

Mean bone density/Group		Control	Study	*P* value^1^	Effect size
Baseline	Mean ± SD	355.52 ± 140.28	429.73 ± 122.12	0.317	0.563
	95% CI	225.77, 485.26	301.57, 557.88		
	Median (IQR)	279.32 (229.06)	485.85 (244.03)		
12th week	Mean ± SD	614.63 ± 172.47	876.96 ± 192.31	0.022*	1.436
	95% CI	455.12, 774.14	674.14, 1078.78		
	Median (IQR)	591.67 (203.75)	888.78 (390.89)		
*P* value^2^		0.018*	0.028*		

*Statistically significant difference at p value < 0.05

*P* value^1^: Mann Whitney U test

*P* value^2^: Wilcoxon Signed Rank test

**Fig. 18:**
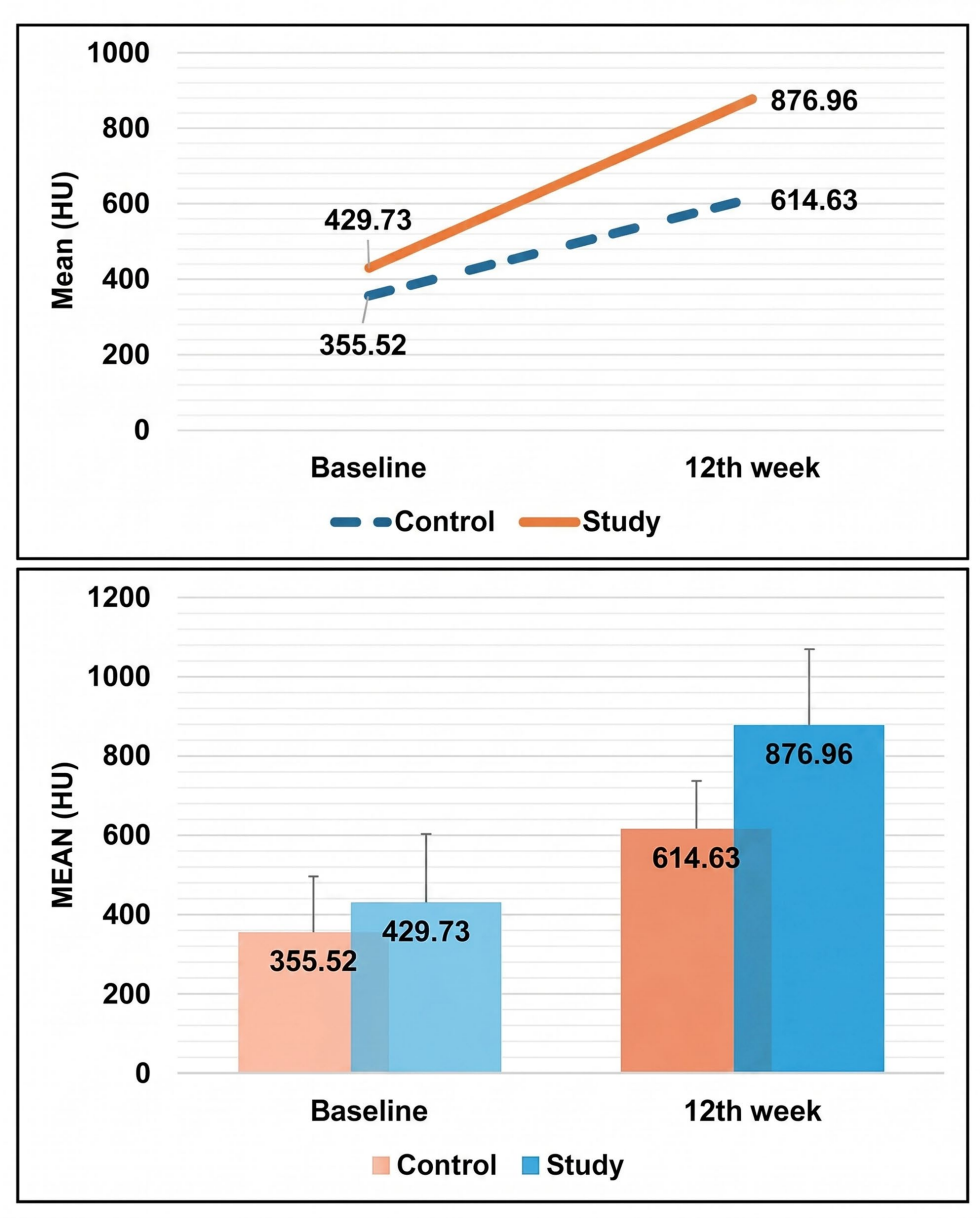
**Comparison between 24 hours and 12 weeks postoperative according to bone density in HU.**

- Immediate postoperative CT scans confirmed satisfactory fracture reduction and alignment in all cases. The mean bone density in the study group was 429.73 ± 122.12 HU, compared to 355.52 ± 140.28 HU in the control group. Follow-up panoramic radiographs at one month demonstrated stable fracture segments.
- After 12 weeks, follow-up CT scans revealed a significant increase in bone density in both groups, with the study group exhibiting higher values (876.96 ± 192.31 HU vs. 614.63 ± 172.47 HU); this difference was statistically significant (*p* < 0.05). Panoramic radiographs on the 12th week also demonstrated trabeculation and bony contours comparable to adjacent healthy bone, indicating complete remodeling. No cases of malunion or nonunion were observed in either group.

## Discussion

This study evaluated the clinical and radiographic outcomes of using low-level laser therapy (LLLT) in combination with titanium-prepared platelet-rich fibrin (T-PRF) for the management of human mandibular fractures. Twelve patients, with a total of fourteen fracture lines, were included. The participants consisted of 10 males (83%) and 2 females (17%), resulting in a male-to-female ratio of 5:1. Mean age was 32.50 ± 12.75 years in the control group, while 29.50 ± 9.99 years in the study group. This male predominance likely reflects higher rates of trauma exposure among males, consistent with global epidemiological patterns of maxillofacial injuries. Such patterns are frequently attributed to greater involvement in violent activities, contact sports, and high-risk occupations. These findings are in agreement with previous studies, including Frimpong et al. [30], who reported a similar male predominance in their analysis of 268 patients, as well as Melek and Sharara [31].

In this study, seven patients had a tooth located in the fracture line. Teeth with grade three mobility were extracted during surgery in three patients after fracture reduction. In the remaining four patients, the teeth were left in place to prevent displacement of the fracture. This clinical dilemma—whether to remove or retain teeth in the fracture line—has been widely discussed in the literature, as in studies by Chrcanovic et al. [32] and Samson et al. [33]. In both cases managed with extraction and IMF, proper healing was observed with no segmental mobility after six weeks, suggesting that LLLT may help accelerate the healing of both hard and soft tissues.

The utilization of 660 nm wavelength irradiation is predicated on its ability to modulate mitochondrial bioenergetics through photon absorption by cytochrome c oxidase. This absorption increases ATP and reactive oxygen species. While possessing shallower penetration than near-infrared (NIR) wavelengths, 660 nm proves biochemically superior in promoting initial bone callus vascularization by stimulating essential angiogenic factors (PDGF-BB, VEGF). Despite depth limitations caused by hemoglobin absorption, bone repair is facilitated via paracrine signaling from superficial tissues [34–40].

Regarding mouth opening, a statistically significant improvement in maximum mouth opening was observed across each group throughout the 12-week follow-up period. By the 12th postoperative week, participants in the study group achieved MMO values comparable to the control group, with no statistically significant intergroup difference observed. These findings align with the MMO recovery patterns reported by Bahari et al. [41] and Raiesian et al. [42] who found that LLLT had no significant effect on trismus. However, it doesn’t come in line with Pavelski et al. [43] results who discovered that LLLT improved trismus across the participants.

The improvement in MMO in each group is most likely attributable to the accurate reduction of bony fragments achieved during ORIF, which restores functional tooth relationships. Mikhail et al. [44] reached similar results concluding that proper bony alignment after surgery improves MMO.

During the follow-up period of this clinical trial, both groups demonstrated notable improvement in postoperative edema, but no statistically significant difference was observed between the two groups. This might indicate that low-level laser therapy did not have a crucial effect on reducing postoperative edema in this context. These results are consistent with Sekerci et al. [45], who found that LLLT did not provide any statistically significant advantages in alleviating pain, postoperative swelling, or trismus following wisdom teeth extraction. Erismen et al. [46] also reported the same findings regarding postoperative edema. However, Baek et al. [47] reported a statistically significant 16.5% reduction in edema in the LLLT group compared to 7.3% in the sham light group (*p* < 0.047), suggesting that LLLT may have promising effects on postoperative edema reduction following facial bone reduction surgery. This result highlights the current variability in the literature regarding LLLT impact on postoperative edema, emphasizing the need for further research to clarify its efficacy across different surgical contexts.

Regarding edema follow-up duration, while acute edema typically resolves within one week following intraoral approaches, the extraoral Risdon incision used in this study involves dissection through the platysma and masseter muscles. Due to the complex vascularity and nature of these tissues, such manipulation leads to more significant reactive edema that may require 10–14 days for complete resolution. To account for this, we selected a three-week follow-up period to accurately differentiate between prolonged physiological edema and pathological swelling (e.g., infection or dehiscence) [48–52].

Pain intensity, quantified using the Visual Analogue Scale (VAS), showed a statistically significant reduction in each group by the second postoperative week. Notably, a significant intergroup difference in pain reduction was observed between the first and second postoperative weeks, with the study group demonstrating superior improvement during this period (*p* < 0.05). However, by the fourth week, pain scores had equalized across both groups. Complete pain resolution was documented in the majority of subjects by the fourth week. This finding aligns with the conclusions of Nesioonpour et al. [53], who reported that LLLT using red (650 nm) and infrared (808 nm) wavelengths shows promise in reducing postoperative pain and the need for painkillers. The results of Bahari et al. [41] and Pavelski et al. [43] also agree with our findings. A systematic review conducted by Seyyedi et al. [54] is also in favor with our findings in pain and states that the LLLT has significantly reduced pain in comparison with the control group and can be used as a promising tool in oral surgeries because of its proven capability in accelerating wound healing and reducing intraoperative pain.

The observed improvement in pain sensation throughout both groups may suggest the efficacy of LLLT and T-PRF in enhancing postoperative recovery, although definitive evidence is still lacking. It is generally presumed that achieving adequate stabilization of bone fragments at the fracture site will resolve patient pain and discomfort, as stated by Zeina et al. [55], making it challenging to attribute pain relief alone to the grafting.

Across the follow-up period, four patients; two in each group, experienced an increase in pain (by 1–2 points on the VAS) in the second week rather than relief. This increase may be strongly related to MRSA infection complication in two cases related to the study group, which aligns with findings by Lee and You [56], who reported increased pain with MRSA infection postoperatively. The two affected cases were managed according to MRSA treatment guidelines outlined by Bhargava et al. [57], and both patients experienced significant pain relief after resolution of the infection.

Only three patients were presented with nerve paraesthesia at admission, with no cases of motor paralysis or facial palsy. As this study focused on posterior mandibular fractures, Chandan S. N. et al. [58] asserted that body fractures (95.9%) had the highest incidence of inferior alveolar nerve dysfunction (IAND), followed by angle fractures (90.1%), highlighting the need for careful monitoring of sensory dysfunction. One case regained normal sensory function immediately after surgery, consistent with Chandan’s findings that most cases showed a significant reduction in severity postoperatively. The other two cases were monitored during healing. Laser stimulation was applied to one case in the study group, which exhibited faster sensory recovery compared to the control group case. This rapid improvement suggests LLLT may play a role in restoring nerve function. This observation comes in line with Fernandes et al. [59] who demonstrated that LLLT effectively improves inferior alveolar nerve paraesthesia following third molar extraction, aligning with this study’s observation of accelerated IAN healing. Diaz et al. [60] further expanded these findings to maxillofacial neuropathies, showing LLLT is a safe and efficacious modality for reducing pain, enhancing neural regeneration, and improving tissue repair.

In our study, the combination of T-PRF and LLLT did not yield significant differences between the two groups in healing scores for soft tissue gap distance, hemostasis, or redness. However, both groups independently showed significant improvement between the second and third postoperative weeks compared to the recordings on the first day.

These findings contradict those of Baek et al. [47], who demonstrated LLLT’s efficacy in wound healing and tissue repair, and Hopkins et al. [61], whose data suggest LLLT promotes partial-thickness wound contraction and indirect healing effects on surrounding tissues. Moreover, both Kaviani et al. [62], and Rashidi et al. [63] reported accelerated healing of chronic wounds (like diabetic foot ulcers) with LLLT, emphasizing its role in soft tissue repair and epithelization.

However, three cases in the study group experienced wound dehiscence five days postoperatively: two due to MRSA infection and one due to the patient not being compliant with postoperative instructions and poor oral hygiene. In these cases, we believe that the frequent LLLT application during the follow-up period accelerated wound closure with fresh epithelium formation despite the fact that these cases faced serious complications, highlighting its potential to enhance wound healing parameters when used with or without T-PRF. This last finding corresponds with Mukhtar et al. [64] who found that Both LLLT and PRF can induce early wound healing. Thorat and Nilesh [65] also reported the same findings regarding wound healing.

Immediate postoperative radiographs yielded a mean bone density of 429.73 ± 122.12 HU in the study group and 355.52 ± 140.28 HU in the control group. By 12 weeks, the disparity between groups widened, with the study group exhibiting a nearly two-fold greater ossification rate (876.96 ± 192.31 HU), while the control group’s mean bone density increased by 73% to 614.63 ± 172.47 HU. Statistical analysis confirmed a significant difference between the two groups. These results align with Elhamshary et al. [66], who found that PRF graft-augmented groups achieved a mean bone density of 890.10 ± 130.55 HU at 6 months, compared to 635.80 ± 94.58 HU in controls. On the same line with us, Cevizcioglu et al. [67] tested the combination of LLLT and PRF in fresh extraction sockets and found that test group 3 (LLLT + PRF) showed histologically under the microscope that approximately 30% new bone trabeculae at the base of the socket, filling 1/3 of the extraction socket, and had higher rates of bone formation among the other groups. Likewise, Kandavalli et al. [24] used a diode laser at variant wavelengths, including the same wavelength used in this study (660 nm) around dental implants and declared that low-level laser therapy aids bone formation, but the wavelength difference had no significant impact. However, Sleem [68] disagrees with these findings and declared that LLLT had no statistically significant effect on bone density around dental implants.

The accelerated maturation of the fracture callus in the study group indicates a more efficient healing trajectory, potentially reducing the risk of nonunion or delayed union. These results underscore the potential of advanced grafting techniques to expedite bone regeneration while improving the quality and mechanical strength of newly formed bone, offering a promising strategy for optimizing patient recovery and long-term functional outcomes. Wang et al. [69] and Elhamshary et al. [66] agree that these findings align with broader evidence demonstrating scaffold-based strategies which optimize the cellular microenvironment for osteogenesis, though long-term comparative studies are still needed to validate these outcomes.

Former in vivo studies have examined this topic histologically at the cellular level under the microscope. Fekrazad et al. [70] investigated the synergistic effects of LLLT (GaAlAs, 810 nm) and mesenchymal stem cells (MSCs) on bone regeneration in rabbit calvarial defects. They reported that while LLLT significantly enhanced bone regeneration, no significant synergistic effect was observed when combined with MSCs. Conversely, Shanei et al. [71] demonstrated in a rabbit calvarial defect model that combining PRF with LLLT improved bone regeneration through increased osteogenesis and reduced fibrosis. However, LLLT alone did not significantly enhance the regenerative process.

Santinoni et al. [72] conducted a systematic review examining low-level laser therapy for maxillofacial bone defects, concluding that postoperative LLLT application enhances bone density. The review further documented LLLT’s anti-inflammatory, analgesic, and wound-healing acceleration properties. However, the authors emphasized that standardized treatment protocols must be established prior to drawing definitive clinical conclusions. Separately, a review by Noba et al. [73] established that LLLT reduces bone healing duration, though protocol standardization remains inconsistent across studies. The mechanism underlying LLLT-mediated tissue healing enhancement remains incompletely understood; however, Kazancioglu et al. [74] propose that tissue absorption of laser light increases mitochondrial activity, local perfusion, adenosine triphosphate (ATP) synthesis, collagen production, and vascular endothelial growth factor release.

In this trial, we measured the bone healing progress rate at 3 months, during the middle of the full mineralization period, to accurately assess the effectiveness of our regenerative interventions. The rationale for this approach is that, as reported by Ramos et al. [75], HU values at almost all fracture sites and in normal bone become indistinguishable by 6, 12, and 18 months postoperatively.

Mean bone density was evaluated using CT imaging. CT scans are considered the gold standard for measuring bone mineralization and are more accurate than cone-beam computed tomography (CBCT). Razi T. et al. [76] reported that HU is the standard scale for measuring CT values. CBCT faces significant limitations in this regard: its grayscale data exhibit reduced reliability due to inherent technical constraints, primarily increased scatter radiation and beam hardening artifacts.

The clinical efficacy of photobiomodulation is fundamentally wavelength-dependent, characterized by a physiological contrast where visible red light (660 nm) drives superficial osteogenesis via localized ATP stimulation, while near-infrared (NIR) wavelengths penetrate deeply to modulate severe pain and edema. Guided by this established contrast, we intentionally utilized the 660 nm wavelength to specifically target bone regeneration. As anticipated, while the treatment successfully stimulated the desired osteogenic pathways, it yielded no statistically significant reductions in pain or edema compared to the control group [77–79].

The adjunctive use of LLLT with T-PRF significantly accelerates bone regeneration. Addressing a critical knowledge gap in the current literature, this finding suggests that LLLT may serve as a valuable method in cases where rapid or enhanced osseous healing is desired, such as in complex maxillofacial defects. Furthermore, it provides promising opportunities for upcoming studies to investigate this combination specifically in compromised populations—such as patients with diabetes, osteoporosis, or steroid-induced suppression. However, the auxiliary use of LLLT in terms of pain reduction, edema resolution, maximum mouth opening (MMO) recovery, and wound healing still needs further investigation.

The findings of this study should be interpreted considering several limitations. First, the sample size was constrained by the scarcity of eligible trauma cases and the strict adherence to inclusion criteria. This relatively-small sample size may limit the statistical power to detect smaller between-group differences and restricts the generalizability of our results to the broader trauma population. Second, regarding study design, blinding of participants and operators was not feasible due to the distinct and obvious nature of the surgical interventions and LLLT application. While we mitigated detection bias for objective outcomes by employing a blinded independent reviewer for radiographic analysis, the lack of blinding for subjective clinical parameters **—** specifically pain, edema, and mouth opening — introduces a potential for performance and measurement bias. Finally, the follow-up period was relatively short which limits our ability to evaluate the long-term sustainability of the treatment effects.

To overcome the limitations of the current study, a multi-center approach would address the scarcity of eligible trauma cases, facilitating larger sample sizes and greater statistical power. Furthermore, to rigorously control the placebo effect and performance bias, future study designs should incorporate sham-controlled LLLT protocols (using inactive probes) to ensure effective blinding of participants. We also recommend extended follow-up periods to assess the long-term stability of the surgical outcomes and the sustained benefits of photobiomodulation on bone healing. Finally, concurrent integration of molecular and histological cellular analyses remains essential to clarify the underlying biological mechanisms.

Given its noninvasive, safe, and patient-friendly profile, further research into LLLT applications in anesthesia and surgical recovery is highly recommended. Continued investigation in these areas will be critical for establishing evidence-based guidelines and maximizing the therapeutic potential of LLLT in maxillofacial and broader surgical practice.

## Conclusions

The adjunctive application of low-level laser therapy with titanium-prepared platelet-rich fibrin significantly enhanced osseous regeneration rates in the study group, yielding higher bone density at 12 weeks postoperatively and greater pain reduction throughout the follow-up period compared to T-PRF alone in the control group. However, both groups demonstrated equivalent improvements in clinical outcomes, including edema resolution, maximum mouth opening recovery, and wound healing, with no statistically significant intergroup differences observed.

## Data Availability

The datasets generated and analyzed during the current study are available from the corresponding author on reasonable request. Due to privacy and ethical restrictions, individual patient data is not publicly available, but de-identified data supporting the findings of this study can be provided upon justified request. All relevant materials, including study protocols and additional documentation, are also available from the corresponding author.

## Footnotes

1 https://clinicaltrials.gov/study/NCT06970379?id=NCT06970379&rank=1.

2 Rosner B. Hypothesis Testing: Two-Sample Inference. In: Fundamentals of biostatistics. 7th ed. Boston: Brooks/Cole. Nelson Education. 2015;269–301.

3 Universität Düsseldorf. G*Power.2019. Retrieved from http://www.gpower.hhu.de/.

4 Tutorial: Analyzing with SPSS Statistics: IBM; [Available from: https://www.ibm.com/docs/en/ias?topic=spss-tutorial-analyzing-statistics.

## Abbreviations

3D: Three-Dimensional
ATP: Adenosine Triphosphate
CBCT: Cone-Beam Computed Tomography
CSH: Clinical Signs of Haemostasis
CSI: Clinical Signs of Inflammation
CSR: Clinical Signs of Re-epithelization
CT: Computed Tomography
EGF: Epidermal Growth Factor
EHS: Early Wound Healing Score
HU: Hounsfield Units
IMF: Intermaxillary Fixation
IQR: Inter Quartile Range
L-PRF: Leukocyte- and Platelet-Rich Fibrin
LLLT: Low-Level Laser Therapy
MMO: Maximum Mouth Opening
MRSA: Methicillin-Resistant Staphylococcus Aureus
NSAID: Nonsteroidal Anti-Inflammatory Drug
ORIF: Open Reduction and Internal Fixation
PDGF: Platelet-Derived Growth Factor
PRF: Platelet-Rich Fibrin
SD: Standard Deviation
T-PRF: Titanium-Prepared Platelet-Rich Fibrin
VAS: Visual Analogue Scale
VEGF: Vascular Endothelial Growth Factor
